# High-efficiency and integrable DNA arithmetic and logic system based on strand displacement synthesis

**DOI:** 10.1038/s41467-019-13310-2

**Published:** 2019-11-26

**Authors:** Haomiao Su, Jinglei Xu, Qi Wang, Fuan Wang, Xiang Zhou

**Affiliations:** 1 Key Laboratory of Biomedical Polymers-Ministry of Education, College of Chemistry and Molecular Sciences, Wuhan University, Wuhan, 430072 China; 2 The Institute of Advanced Studies, Wuhan University, Wuhan, 430072 China; 3 Key Laboratory of Analytical Chemistry for Biology and Medicine (Ministry of Education), College of Chemistry and Molecular Sciences, Wuhan University, Wuhan, 430072 China

**Keywords:** Synthetic biology, DNA computing

## Abstract

Powerful information processing and ubiquitous computing are crucial for all machines and living organisms. The Watson-Crick base-pairing principle endows DNA with excellent recognition and assembly abilities, which facilitates the design of DNA computers for achieving intelligent systems. However, current DNA computational systems are always constrained by poor integration efficiency, complicated device structures or limited computational functions. Here, we show a DNA arithmetic logic unit (ALU) consisting of elemental DNA logic gates using polymerase-mediated strand displacement. The use of an enzyme resulted in highly efficient logic gates suitable for multiple and cascaded computation. Based on our basic single-rail DNA configuration, additional combined logic gates (e.g., a full adder and a 4:1 multiplexer) have been constructed. Finally, we integrate the gates and assemble the crucial ALU. Our strategy provides a facile strategy for assembling a large-scale complex DNA computer system, highlighting the great potential for programming the molecular behaviors of complicated biosystems.

## Abstract (web summary)

Current DNA computational systems are constrained by integration efficiency, device structures and limited functions. Here the authors design a DNA arithmetic logic unit that uses polymerase-mediated strand displacement.

## Introduction

The beautiful intricacies of living organisms rely on information processing and ubiquitous computing at the molecular level. Constructing biomolecular information processing systems with ingenious functions may advance our understanding of life. While most biological systems store their genetic information in DNA, enzymes endow life processes with high efficiency. Many DNA computational systems have been developed with or without enzymes to explore the potential of molecular computer systems^1^.

The first prototype DNA computer was presented by Leonard Adleman in 1994 and was used to solve the seven-city Hamiltonian path problem with DNA ligase and polymerase^2^. Since then, deoxyribozyme^3^, restriction endonuclease^4,5^ and toe-hold exchange^6^ have been applied to build DNA computational devices. Among these, the toe-hold exchange strategy shows the most promising properties and successfully performed the first large-scale cascaded logic computation with DNA based on a seesaw gate motif^7^. Furthermore, carefully designed DNA seesaw circuits have been applied in neural network computations^8,9^.

The strand displacement DNA synthesis was carried out with an upstream primer and DNA polymerase that could achieve strand displacement. Along with the primer extension, the new synthetic DNA strand could displace and release the downstream complementary strand. Based on this property, many DNA amplification methods, including strand displacement amplification (SDA)^10,11^, loop-mediated isothermal amplification (LAMP)^12^ and rolling circle amplification (RCA)^13^, have been developed. Besides, strand displacement synthesis has been used to build dynamics DNA circuits by coupling with restriction and nicking enzymes^14^. The intrinsically sustainable and highly efficient enzyme-mediated strand displacement method inspired us to implement logic operations based on this enzymatic approach.

Here, we design DNA logic gates with high efficiency, compact structure and an ability to build cascade circuits based on polymerase-mediated strand displacement synthesis. We build dual-rail DNA logic gates by paralleling a single-rail AND gate and a single-rail OR gate to construct any logical expression. In addition, we successfully construct a 1-bit full adder and a 4:1 multiplexer with our DNA logic gates. Finally, we integrate the 1-bit full adder and the 4:1 multiplexer and obtain a crucial DNA ALU, which is a multifunctional device, that can be used to directly assemble the central processing unit (CPU) of digital computers. The DNA ALU we construct has 16 equivalent logic gates inside and consists of 27 DNA species and 74 DNA strands. The successful construction of the sophisticated DNA ALU reveals the powerful ability to construct a DNA computer system for our design.

## Result

### Implement basic logic gates with DNA strand displacement

Although DNA computational systems have undergone many advances, they are only used in simple combinations with logic gates and are far from being highly functional bio-computational devices; these limitations need to be addressed before their practical applications can be envisaged. To build a highly efficient and sophisticated DNA computing system, we believed three issues should be considered: (I) keeping the input and output in the same form, making it suitable for a cascade circuit; (II) constructing logic gates with a compact DNA configuration; and (III) developing a highly efficient computational system. As previous reports have not satisfactorily met these criteria, we designed a DNA bio-computer system with strand displacement DNA synthesis to address these considerations.

**Fig. 1:**
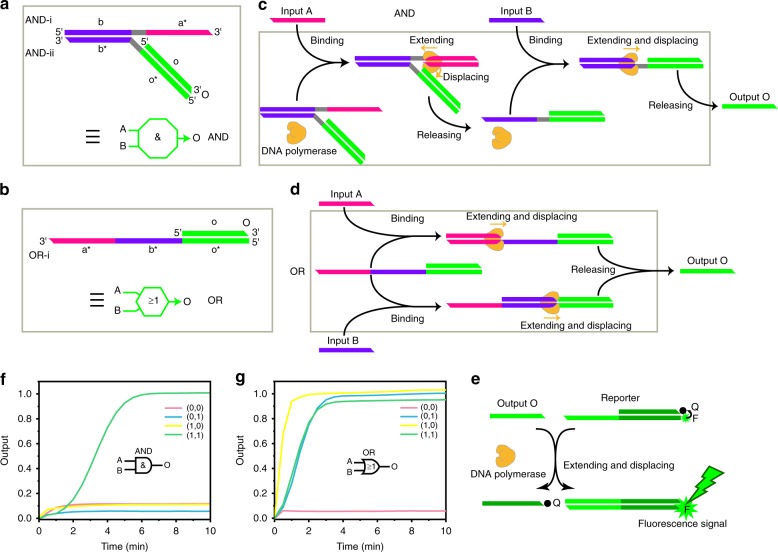
Construction of AND and OR gates with DNA polymerase and their performance. **a**, **c** Right: structure of the AND and OR gates. The capital names are the sequence names; the lowercase names refer to the elementary sequences and asterisk indicates a complementary sequence. Each elementary sequence contains 18 bases. The gray parts are 4-mer spacer sequences used to reduce steric hinderance. The sequences of inputs A and B are the same as a and b. Left: abstract diagram of the AND and OR gates. The regular octagon shows the main structure of the DNA components; the two bold lines on the left indicate the binding sites of the two inputs in the DNA component; the vector line on the right indicates the potential output. **b**, **d** Mechanism of the AND and OR gates. **e** The fluorescent reporter used to visualize the devices. The letter Q denotes the quencher, and F denotes the fluorophore. **f**, **g** Reaction kinetics of the AND and OR gates with all possible combinations of inputs. The reaction was performed with 3.2 U Bst polymerase (large fragment) and TRI at 35 °C. The curve was plotted by transferring the cycle value into the reaction time. The outputs were normalized to the relative fluorescence unit (RFU) values in the FAM channel with the highest signals. The original signals are plotted in Supplementary Fig. 3. The sequences of the DNA strands are listed in the Supplementary Table 2.

We started designing our DNA bio-computer with a characteristic AND gate that consisted of three strands: AND-i, AND-ii, and O (Fig. 1a). Input A binds to the elementary sequence a* in strand AND-i. Then, polymerase-motivated extension of input A would displace strand AND-ii, exposing the binding site of input B. Then, input B could be extended to release output O (Fig. 1b). As the binding site of input B is blocked by strand AND-i, the constructed DNA device can only release output O in the presence of both inputs (A and B), which is consistent with the requirement of an AND gate. Our modular and compact DNA bio-computer system could be easily altered to build an OR gate with two strands: OR-i and O (Fig. 1c). When the binding sites of inputs A and B are both unblocked, the presence of either A or B will directly result in the hybridization of strand OR-i, releasing O (Fig. 1d). Importantly, the coherent form of input and output strands facilitated the use of our logic system to build cascade circuits (1st requirement). In addition, the simple configuration makes it compact (2nd requirement). To better present the cascaded logic gates below, we abstracted our AND and OR gates as regular octagons and a regular hexagon with two blunt lines on the left for the binding sites of two inputs as well as a vector line on the right representing the output (Fig. 1a, b). Finally, we used a reporter that quenches fluorescence (Supplementary Fig. 1) to visualize our devices (Fig. 1e) because the released output O could displace the strand with the quencher to recover the fluorescence of the transduction element.

By annealing the DNA components of two gates, we successfully assembled the target structures (Supplementary Fig. 2). After purifying the strands with native polyacrylamide gel electrophoresis (PAGE), we tested our logic gates and visualized the results with real-time PCR as well as PAGE followed by gel imaging. All the results showed that the DNA components could properly perform the desired logic operation (Fig. 1f, g and Supplementary Fig. 3). Moreover, the short computation time (~6 and 3 min for AND and OR operations, respectively) confirmed the high efficiency of the system, which meets the 3rd requirement. We also optimized the reaction conditions (Supplementary Figs. 4–6). These results showed varied signal leakage with different DNA polymerases, which can be attributed to the poor specificity of the enzymatic biotransformation. We chose Bst polymerase because it showed the lowest leakage and a satisfactory transformation efficiency.

**Fig. 2:**
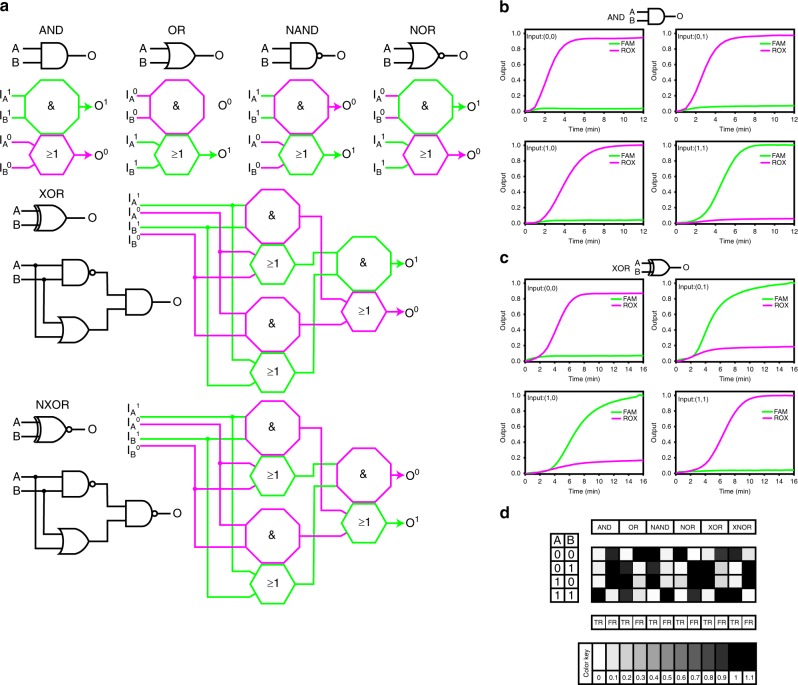
Construction of six basic dual-rail systems and their performance. **a** Construction of the AND, OR, NAND, NOR, XOR, and XNOR dual-rail gates. The upper regular octagon shows a single-rail AND gate, and the lower regular hexagon shows a single-rail OR gate. The green and magenta colors mean defined TRUE and FALSE signals, respectively. Superscripts 1 and 0 also indicate the TRUE and FALSE inputs and outputs, respectively. When computations were performed, a set of input strands must be adder, e.g., inputs (1, 0) means adding inputs I_A_^1^ (TURE) and I_B_^0^ (FALSE). Detailed structures can be seen in Fig. S12. Reaction details of AND and XOR gates can be seen in Supplementary Figs. 7 and 8. **b**, **c** Reaction kinetics of the dual-rail AND and XOR gates with all possible combinations of inputs. The reaction was performed with 3.2 U Bst polymerase (large fragment), TRI and FRI at 35 °C. The curve was plotted by transferring the cycle value into the reaction time. The outputs were normalized to the RFU values in the FAM and ROX channels with the highest signals. The FAM and ROX signals correspond to the TRUE and FALSE returns, respectively. Sequences of the DNA strands are listed in the Supplementary Information. **d** Summary of all the outputs computed by the six basic logic gates constructed from DNA.

In addition to the AND and OR logic gates, the NOT operation is indispensable for building a functional complete set, with which any logic computation can be constructed. Because the downstream NOT gate does not wait to produce the output TRUE before a signal is generated upstream in use-once circuits, the NOT gate is difficult to implement^7^. We developed our logic gates into a dual-rail AND gate, which could achieve a NOT operation by reversing the definition of the strands, by constructing a single-rail AND gate and a single-rail OR gate as well as defining a set of FALSE input/output (see Fig. 2a, and more details could be found in Supplementary Figs. 7 and 12). It should be noted that now the 0 means adding/releasing a FALSE input/output strand and there were four entrances for different inputs in the dual-rail gates. In addition, we added a false reporter (FRI) to detect false signals in the ROX channel. Figure 2b. shows the favorable and efficient performance of our dual-rail AND gates (operation time <8 min). PAGE further confirmed our results (Supplementary Fig. 9a). In addition to dual-rail AND gates, we also constructed five more basic logic gates with the same inputs and outputs by rearranging the elementary sequence (XOR, NAND, and NOR) or adding more elementary sequences and DNA components (XOR and XNOR; details can be found in Supplementary Fig. 12). Notably, the facile and formulaic construction demonstrated that this system is well suited to the construction of logic gates. We tested the constructed gates, and they all showed reliable and highly efficient performances (Fig. 2c, d and Supplementary Figs. 9–11). Moreover, the XOR and XNOR gates were assembled with cascaded single gates, which preliminarily confirmed that complex DNA digital systems can be constructed.

### Constructing digital devices with DNA logic gates

**Fig. 3:**
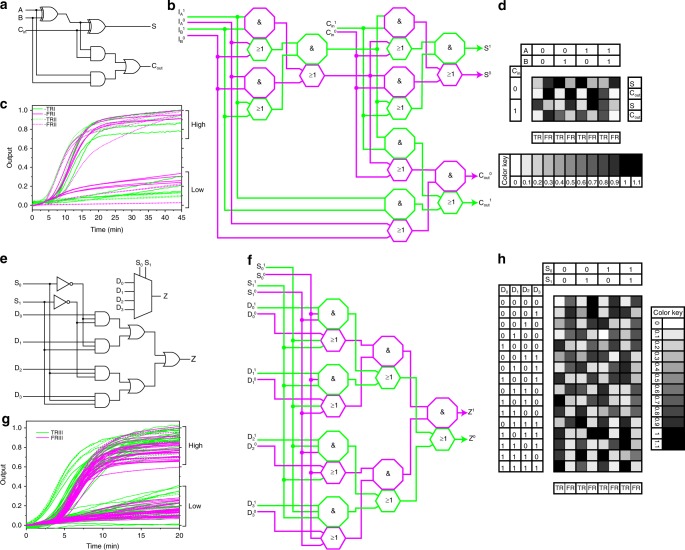
Construction of the full adder and 4:1 multiplexer from our DNA logic gates. **a**, **e** A typical construction of the full adder and the 4:1 multiplexer with the digital logic circuit. D_0_–D_3_: inputs; S_0_ and S_1_: select signals; Z: output. **b**, **f** Constructing a full adder and the 4:1 multiplexer with DNA logic gates. The sequences of the DNA strands are listed in the Supplementary table 2. **c**, **g** Summary of all the outputs computed by the DNA full adder and 4:1 multiplexer. **d**, **h** Reaction kinetics of the full adder and the 4:1 multiplexer with all possible combinations of inputs. For the full adder, the reaction was performed with 12 U Bst polymerase (large fragment), TRI, FRI, TRII, and FRII. The outputs were normalized to the RFU values in the FAM, ROX, HEX, and Cy5 channels with the highest signals. For the full adder: The TRI (FAM) and FRI (ROX) signals correspond to TRUE and FALSE returns of S, respectively. The TRII (HEX) and FRII (Cy5) signals correspond to the TRUE and FALSE returns of C_out,_ respectively. For the 4:1 multiplexer, the reaction was performed with 6.4 U Bst polymerase (large fragment), TRIII and FRII. The outputs were normalized to the RFU values in the FAM and ROX channels with the highest signals. The TRIII (FAM) and FRIII (ROX) signals correspond to TRUE and FALSE returns, respectively. The reaction kinetics of the individual gates can be seen in Supplementary Figs. 14 and 19.

With the basic components in hand, we then started exploring some digital devices. An adder is a fundamental component containing many arithmetic units and is found in almost all computers. A full adder is used to add two addends (A and B) and the result from a previous parallel adder (C_in_). Computations with a full adder produce two outputs: a sum (S) and an output for the next adder (C_out_; Fig. 3a shows a typical construction). We assembled a full adder with our DNA logic gates (Fig. 3b and more details can be found in Supplementary Fig. 13). The system was built with only 18 DNA species consisting of 45 different DNA strands, while similar device typically requires 72 DNA species and 126 different DNA strands when constructed based on a classical seesaw DNA logic system^7^. The simple configuration of our system could make DNA biocomputers more accessible and operationally simpler. In addition, we added a pair of reporters (TRII and FRII) to simultaneously detect the C_out_ signals of the Hex/Cy5 channels. As expected, our full adder performed as designed (Fig. 3c, d and Supplementary Fig. 14). Although the full adder was much more complicated than single logic gates, the computation was still finished in 20 min.

Then, we assembled a multiplexer to build a multifunctional DNA computer. The multiplexer, also called the data selector, is a device that can select one input from multiple inputs and deliver it into output. The multiplexers make it possible to realize multiple functions in one device, which is important for the complicated processing systems that are wildly used in combinational circuits, e.g., processors^15^. The 4:1 multiplexer has four inputs, one output, and two select signals (Fig. 3e shows a typical construction). Four combinations of select signals [(0,0), (0,1), (1,0), and (1,1)] deliver different inputs (D_0_–D_3_) to the output. To obtain a more compact construction, we integrated two AND gates into a 3-input AND gate by simply adding a strand to a single-rail AND gate and an elementary sequence to a single-rail OR gate (Supplementary Fig. 15). We assessed the system with PAGE and qPCR and found it was assembled as designed and worked property (Supplementary Figs. 14 and 17). Then, we assembled a DNA 4:1 multiplexer with our DNA components (Fig. 3e, more details can be found in Supplementary Fig. 18). The system contained 14 DNA species consisting of 39 different DNA strands. We tested all input combinations and found that the system could convert the inputs into the correct outputs (Fig. 3g, h and Supplementary Fig. 19) with a relatively short reaction time (~20 min).

### Assembling DNA ALU with DNA logic gates

**Fig. 4:**
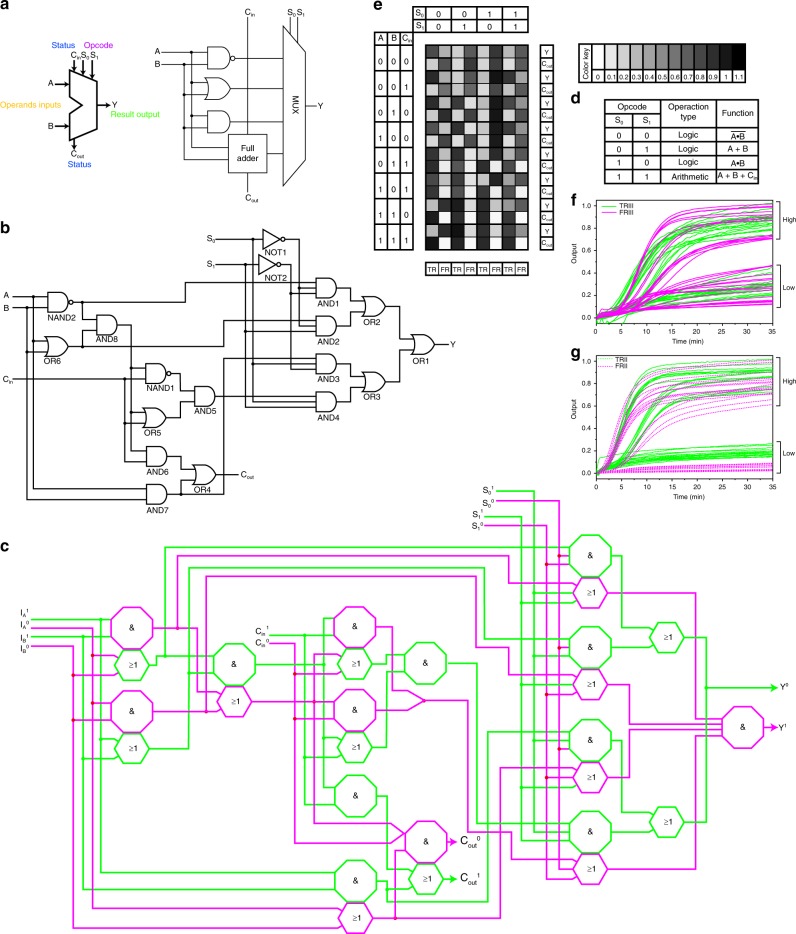
Construction of DNA ALU with our DNA logic gates. **a** Abstract diagram of a typical ALU. **b** A typical construction of a 1-bit ALU with a digital logic circuit. A and B: inputs; C_in_: carry in; S_0_ and S_1_: select signals; Y: output; C_out_: carry out. **c** Assembling the 4:1 multiplexer with DNA components. The sequences of the strands are listed in the Supplementary Table 2. The details of the integrated logic gates are shown in Fig. S20. **d** Function table of the DNA ALU. **e** Summary of the outputs of the ALU. **f**, **g** Reaction kinetics of the ALU with all possible combinations of inputs. The reaction was performed with 12 U Bst polymerase (large fragment), TRIII, FRIII, TRII, and FRII. The curve was plotted by transferring the cycle values into the reaction time. The outputs were normalized to the RFU values in the FAM, ROX, HEX, and Cy5 channels with the highest signals. The TRIII (FAM) and FRIII (ROX) signals correspond to the TRUE and FALSE returns of Y, respectively. The TRII (HEX) and FRII (Cy5) signals correspond to the TRUE and FALSE returns of C_out,_ respectively. The reaction kinetics of the individual gates can be seen in Supplementary Fig. 22. The sequences of the DNA strands are listed in the Supplementary Table 2.

Inspired by the favourable performance of the full adder and 4:1 multiplexer, we constructed a 1-bit ALU by integrating these well-established logic devices. The ALUs are the principal components of a CPU and the heart of most computer systems^16^. The ALU performs different operations when different opcodes are added, so we can achieve multiple functions in one device (Fig. 4a, b). As far as we know, no DNA ALU has been realized because the information transfer in the ALU is constrained by the sophisticated configuration and poor efficiency. Hence, we introduced additional integrated gates instead of directly combining the full adder and multiplexer to maintain the efficiency of the system (Fig. 4c. and Supplementary Fig. 20). Then, we prepared a 4-function ALU consisting of 27 DNA species and 74 DNA strands (Fig. 4c). Furthermore, the assembled ALU worked properly and efficiently with three logic functions (NAND, OR, and AND) as well as one arithmetic function (full adder) (Fig. 4d, f and Supplementary Fig. 22). Although the ALU had relatively high leakage in output Y, the TRUE and FALSE outputs are still easily distinguished.

## Discussion

The leakage is the main challenge in our system, especially when the size scales up. The limited purity of commercial chemosynthetic DNA strands and DNA components introduced inconspicuous primary leakage. We performed some optimizations to reduce this leakage (Supplementary Figs. 4, 6, 23, and 24). However, as the size of the circuits scale up, the inconspicuous leakages of each individual gate were integrated together into a significant final leakage. We expected that a high-affinity competitor, e.g., peptide nucleic acid, to the binding site of outputs could eliminate the leakage further. Another challenge is the fast-growing DNA–DNA cross interactions when the system scales up. In our case, we only considered the cross interactions that would result in nonspecific extension. However, the complicated cross interactions may slow desired interactions when further scaling up. Hence, a fast and automatic prediction and design method is indispensable in further studies.

Although there are some formal resemblances between our method and enzyme-free DNA strand displacement, the basic strategies are distinct. Enzyme-free logic gate systems integrated different inputs without bias and compared the sum with different thresholds to obtain different logic computations, which is actually a mathematic solution^7^. Hence, excess input can trigger the AND gate. In contrast, our method used different architectures (tandem or parallel binding sites) to carry out strictly logical computations, which is actually a physic solution. Meanwhile, our system offered ~50-fold efficiency while only requiring ~1/3 of the strands and 1/4 of the components compared with the typical enzyme-free system (Supplementary Table 1). Moreover, even the specially designed enzyme-free single-rail half adder required the same number of strands that we used to build a dual-rail half adder^17^. Finally, the introduction of enzymes could offer additional space to improve efficiency and fidelity by enzyme screen or engineering^18^. At the time we secondly revised our manuscript, another polymerase-mediated DNA logic circuits with completely different design of logic gates published on Nature Nanotechnology^19^. Their work constructed the AND gate with 2 strands and the OR gate with 3 strands, which means we used equal strands to construct the basic dual-rail logic gates. However, our design show more powerful on constructing complex logic circuits as our logic gates are integrable. For example, while we can simply integrate 2 AND gates into a 3-input AND gate, their architecture did not support the 3-input logic gates. Significantly, their leakage is lower than us as they used single strands to build the gates. We believe the obvious compatibility between our design and theirs would promote the development of DNA computers together.

In summary, we developed a highly efficient DNA logic system that is integrable, modular and compact by using an enzyme. In addition, we succeeded in assembling complicated digital circuits such as an ALU with high efficiency. With the functionally complete set and high efficiency, our system in principle has the ability to construct more complicated logic or arithmetic circuits. In particular, we showed that we can manipulate an intricate molecular system with nucleic acid, which is the carrier of hereditary information in a living system and is used as the foundation of many bio-applications. Thus, our DNA computer system may have a broad application for providing a platform for analysis and controlling molecular systems. On the one hand, the nucleic acids, e.g., microRNAs^20^ as well as piRNAs^21^, are the carriers of information from many organisms and can directly be used as inputs in our molecular computer system. Other molecules can also be transformed into DNA inputs by applying aptamers and ribozymes^22^. On the other hand, the outputs of our system may be directly used to control DNA molecular machines, e.g., DNA walkers^23^ and DNA cargo-sorting robots^24^. Furthermore, the RNA output devices, such as functional ribozymes^25^ would be easily achieved by coupling with in vitro transcription.

## Methods

### DNA sequences

All the DNA sequence used in our paper are list in Supplementary Table 2.

Design of logic gates and reporting probes: As our logic gates assembled with elementary sequences, the design of logic gates and reporting probes were based on elementary sequence levels. The first step in constructing DNA logic gates is obtaining a set of 18-mer elementary sequences. The requirements of the elementary sequences mainly agree with the requirements of the primers: (a) 40–60% GC content, (b) avoiding stable secondary structures (ΔG > −3 kcal mol^−1^), (c) neither the 3′ or 5′ tail being complementary to the internal sequence, and (d) avoiding the last five bases in the 3′ tail being complementary to any sequence in this set. Here, we used the NUPACK web application to obtain the minimum free energy structure and free energy of the strands^26^. Then, the logic gates were assembled from the elementary sequences according to Fig. 1a, c and Supplementary Fig. 10. All the assembled sequences should be checked to confirm that no stable secondary structures were generated. The relative positions of the inputs in one structure can be exchanged to avoid forming stable secondary structures (e.g., exchanging the position of elements a and b in the structure of single-rail OR gates). If it does not work, the elementary sequence should be replaced with a new one, and the assembled structure should be rechecked.

Assembling the logic gates and reporting probes: All the sequences of the logic gates were purified by reversed-phase chromatography, and all the input sequences were purified by PAGE except the strands used in optimization experiments. All the sequences for reporting probes were purified by HPLC. All oligonucleotides were purchased from Genscript (Nanjing). The powder of oligonucleotides was dissolved in 1 × Tris/Mg^2+^ buffer (50 mM Tris, 6 mM MgCl_2_, pH 8.0) and the concentration was accurately determined in NanoDrop 2000 by inputting the corresponding sequence. All the gates strands were annealed together at concentrations of ∼50 μM by first holding at 95 °C for 10 min and then cooling to 4 °C at a rate of 0.5 °C/min. This step was performed with a Bio-Rad T100™ Thermal Cycler. After adding glycerol to 6% (v/v), the solution was purified using 10 or 12% native PAGE of 1.5 mm thickness, which was run for 4 h at 250 V in 1 × TBE buffer (89 mM Tris, 89 mM boric acid, and 2 mM EDTA) at room temperature. The gel bands were clearly visualized under UV light, excised from the gels, crushed and soaked in 1 × TBE buffer overnight at room temperature. The crushed gel particles were removed from the solution by centrifugation at 12,000 × *g* for 5 min, and the supernatant was concentrated to 25 μL with a 10 K Amicon^®^ Ultra-0.5 centrifugal filter. Finally, after three rounds of dialysis against 1 × Tris/Mg^2+^ buffer, the solution was stored at room temperature. The absorbance at 260 (OD_260_) of the recovered DNA components was measured with NanoDrop 2000. The extinction coefficients (e, units: L mole^−1^ cm^−1^) were the recovered DNA components calculated with the formula e = Σe_i_−3200*N_AT_ −2000*N_GC_, where N_AT_ and N_GC_ are the number of AT pairs and GC pairs in the double-stranded domain, respectively^7^. The concentration (c, units: μM) of the recovered DNA components was calculated as c = OD_260_/e × 10^6^.

The fluorescent reporters were annealed together at 4 μM with 20% excess of the strands abeled quencher in 1 × Tris/Mg^2+^ buffer. The annealing protocol was the one used for annealing gates. After annealing, the fluorescent reporters were stored in the dark at 4 °C.

Performing computation of DNA devices: All reactions were performed with a Bio-Rad CFX96 Touch™ Real-Time PCR Detection System. For experiments involving four fluorophores, the protocol was equipped with four individual scanning channels (FAM, ROX, HEX, and Cy5). Otherwise, the typical protocol involved two channels (FAM and ROX). First, to prepare the reaction solutions more accurately, all stock DNA component solutions were pre-diluted to 5 μM, and all stock input strands solutions were prediluted to 10 μM. Typically, 1 pmol of the DNA species (recovered DNA components and input strands) was used in the reaction, and the reaction volume was 10 or 20 μL (the corresponding final concentration was 100–50 nM) unless specifically stated. However, when the DNA logic gate have multiple downstream logic gates, their quantities should be multiplied by the number of downstream logic gates, for instance the input strands were 2 pmol as they input two logic gates in the dual-rail XOR gates. The reaction mixtures were prepared based on the different enzymes. Typically, we used Bst DNA polymerase, large fragment (New England Biolabs), and the reactions were performed in 1 × ThermoPol buffer containing 4 mM of MgSO_4_, 0.31 mM each dNTP. The unit of enzyme Bst DNA polymerase was described in the figure captions for every experiment. The reaction mixtures were prepared at room temperature, and the protocol was run at 35 °C with a run time of 20 s. Then the system was photographed (each cycle takes ~10 s). To normalize the curves, the first ten cycles were run without the enzyme to measure the background. After ten cycles, the protocol was paused, and the enzyme was added to the reaction tube. Once the lid was closed, the protocol was resumed.

Data normalization: The qPCR data were first normalized to cycles 7–9 to obtain the RFU curves by the CFX Manager software. Then, the means of the RFU values in cycles 7–9 in all parallel samples were used to obtain the background (F_0_), and the mean of the RFU values (F) in the last three cycles of the samples that gave the highest TRUE signals were considered the full output (1). Then, the background was subtracted from all RFU values and they were divided by F to obtain the normalized data.

PAGE analysis: The assembled or purified DNA components and products of the computation were analysed with 12% native PAGE of 1.0 mm thickness. The DNA components were stained with GelRed^®^ and scanned with ChemiDoc XRS + (Bio-Rad Laboratories) in a Gel Red channel. The reaction products were directly scanned with a ChemiDoc MP imaging system (Bio-Rad Laboratories) in FAM and ROX channels.

### Reporting summary

Further information on research design is available in the Nature Research Reporting Summary linked to this article.

## Supplementary information

Reporting Summary

Supplementary Information

## Data Availability

The uncropped gels of Supplementary Figs. 2, 3, 9a, 9c 10b, 10d, 11a, 11c, 16, 24 are available in the Source Data file. All other data supporting the findings of this study are available within the Article and its Supplementary Information, or from the corresponding author upon reasonable request.

## Source Data

Source Data
