# Supplementary material for: High-efficiency and integrable DNA arithmetic and logic system based on strand displacement synthesis
Source: Nat Commun. 2019 Nov 26;10:5390. doi: 10.1038/s41467-019-13310-2 (PMC6879481; doi:10.1038/s41467-019-13310-2)
Supplement: Supplementary file 2 — Supplementary Information [file 41467_2019_13310_MOESM2_ESM.pdf]

Supplementary Information

High-efficiency and integrable DNA arithmetic and logic system based on strand displacement synthesis

Su *et. al.*

Supplementary Figures

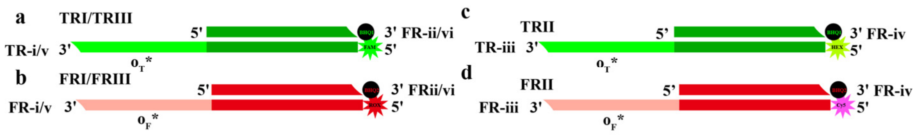

Supplementary Figure 1 Structure of the reporting probes; the capital letters indicate the sequence name; lowercase letters stand for the sequence elements and * indicates complementary sequences. The released O sequence will anneal with reporting probes, and the extension of O will release the quenching sequence and recover the fluorescence of the reporter. **a.** TRI/TRIII: the 5' end of TR-i/v was labelled with FAM, and the 3' end of TR-ii/v was labelled with BHQ1. **b.** FRI/FRIII: the 5' end of FR-i/v was labelled with ROX, and the 3' end of FR-ii/v was labelled with BHQ2. **c.** TRII: the 5' end of TR-iii was labelled with HEX, and the 3' end of TR-iv was labelled with BHQ1. **d.** FRII: the 5' end of FR-iii was labelled with ROX, and the 3' end of FR-iv was labelled with Cy5. The O_T and O_F sequence elements were different for different reporters. TR and FR are abbreviations for the true reporter and false reporter, respectively. The sequences of the strands are listed in the Supplementary Table 2.

lane	1	2	3	4	5	6	7	8	9	10	11	12	13	14	15	16
AND-I	+			+		+			+							
AND-II		+		+	+	+			+	+						
OR-I											+		+	+	+	+
OP			+		+	+			+	+		+	+	+	+	+
A							+		+					+		+
B								+		+					+	+

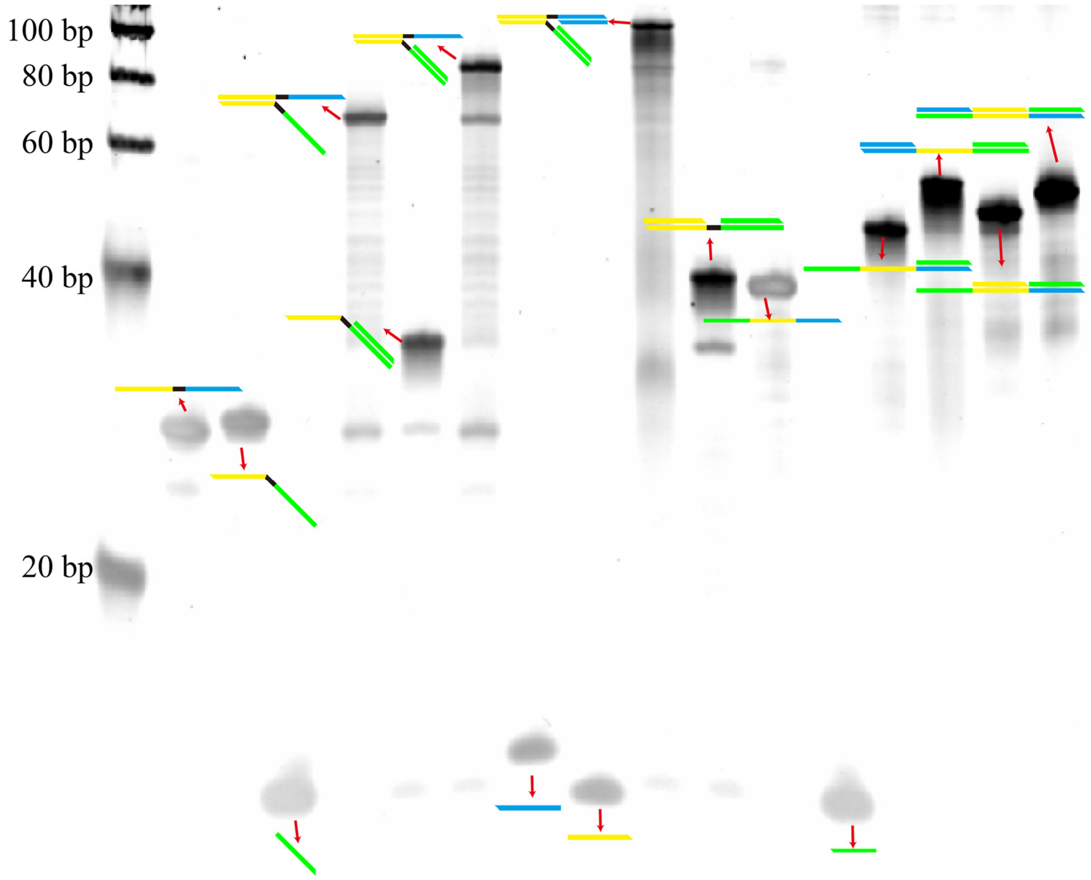

Supplementary Figure 2 Assembling of the logic gates and their binding to the input strands. Each lane was annealed with the indicated strands. The PAGE results show that the designed structure was successfully assembled, and the binding of the input strands were achieved. Source data are provided as a Source Data file.

a

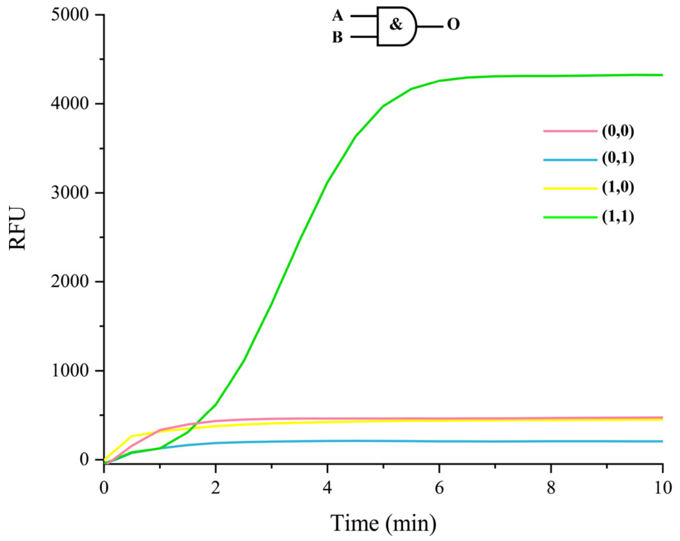

b

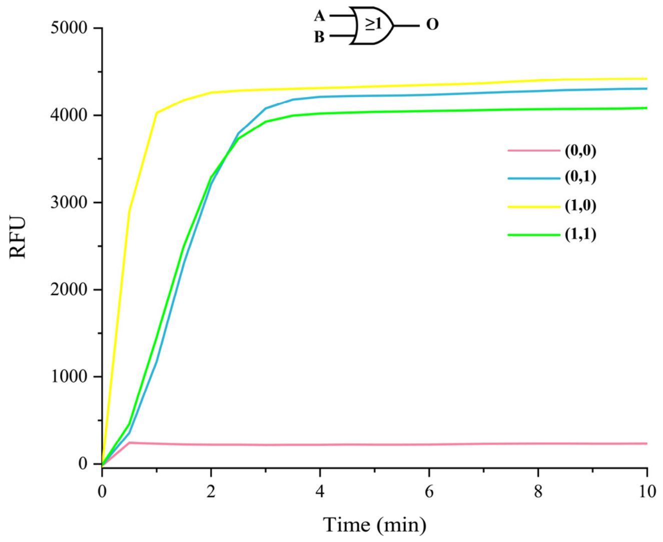

c

Lane	1	2	3	4		5	6	7	8
Gate	OR					AND			
A	0	0	1	1		0	0	1	1
B	0	1	0	1		0	1	0	1
Output	0	1	1	1		0	0	0	1

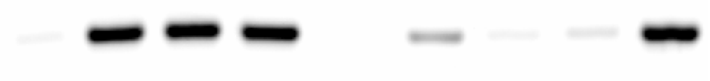

Supplementary Figure 3 **a.** Unnormalized reaction kinetics in Figure 1e. **b.** unnormalized reaction kinetics in Figure 1f. **c.** PAGE analysis of the products produced by the logic gates. As the probe had been quenched, the recovered fluorescent bands indicated the release of the output strand and corresponded to the true output. Source data are provided as a Source Data file.

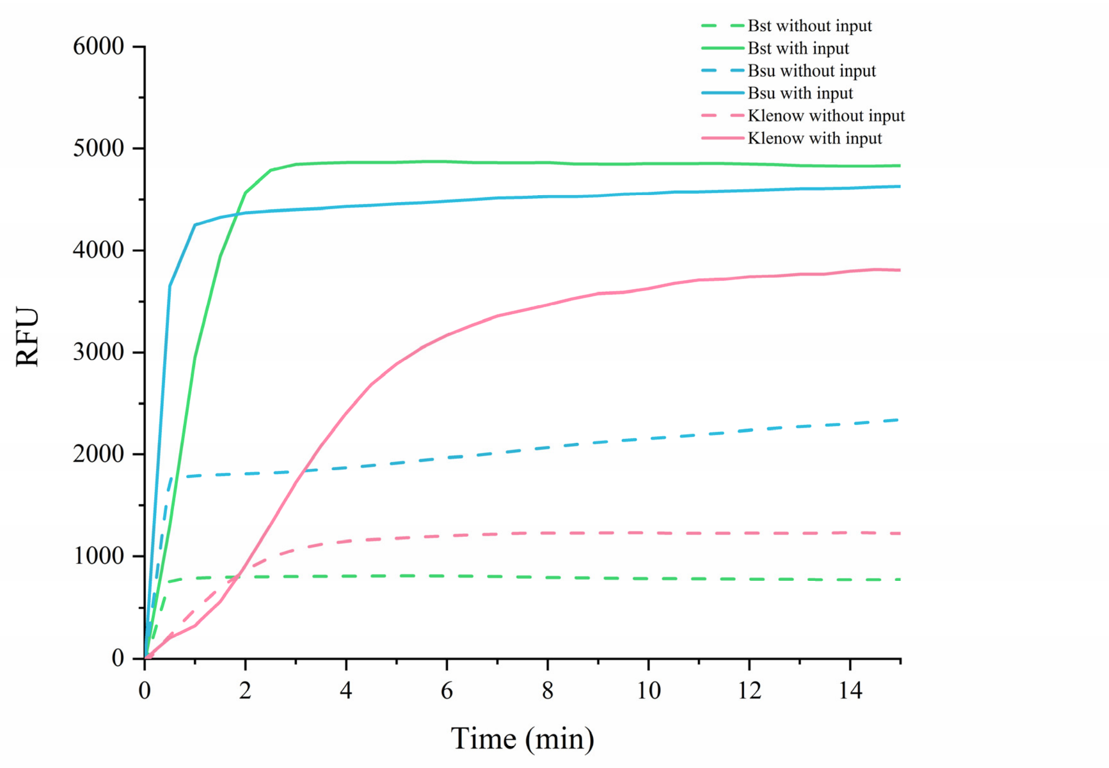

Supplementary Figure 4 The performance of 3 different strands displacing DNA polymerase on an AND gate. While the Bsu polymerase gave a higher reaction speed, it showed a relatively high leakage. Hence, we chose Bst polymerase as the enzyme for subsequent experiments.

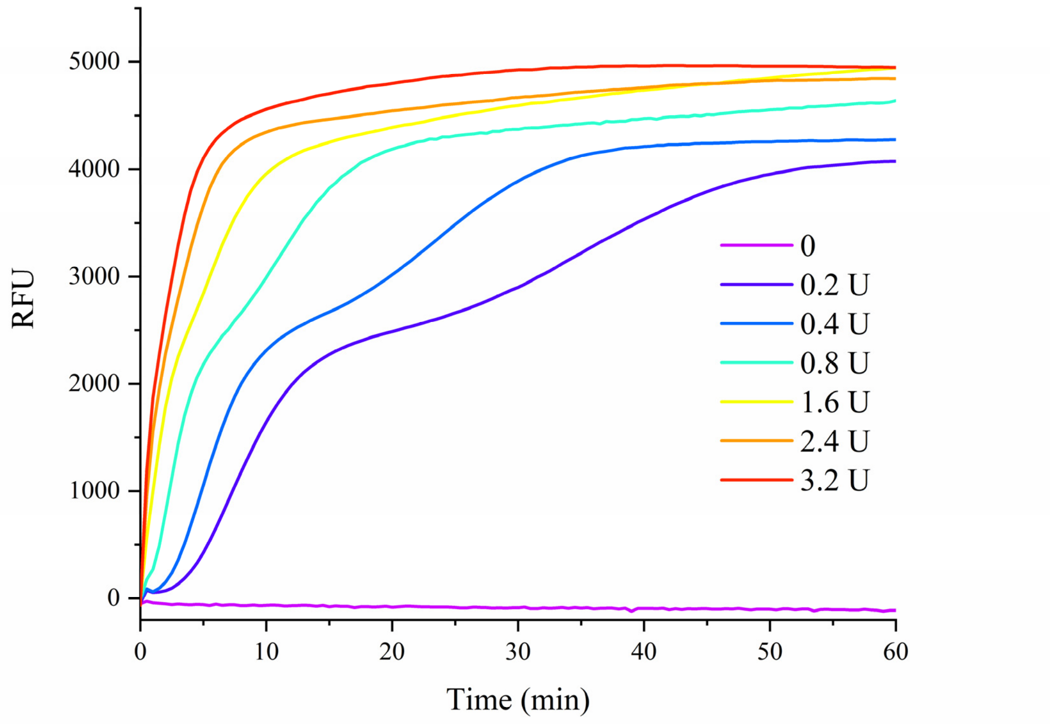

Supplementary Figure 5 Optimization of the enzyme unit (Bst DNA polymerase) for a single-rail AND logic gate. From the results, we chose 3.2 U for performing computations with one logic gate. The enzyme units for other complicated DNA devices for performing other operations were tested based on this result (data not shown in the paper).

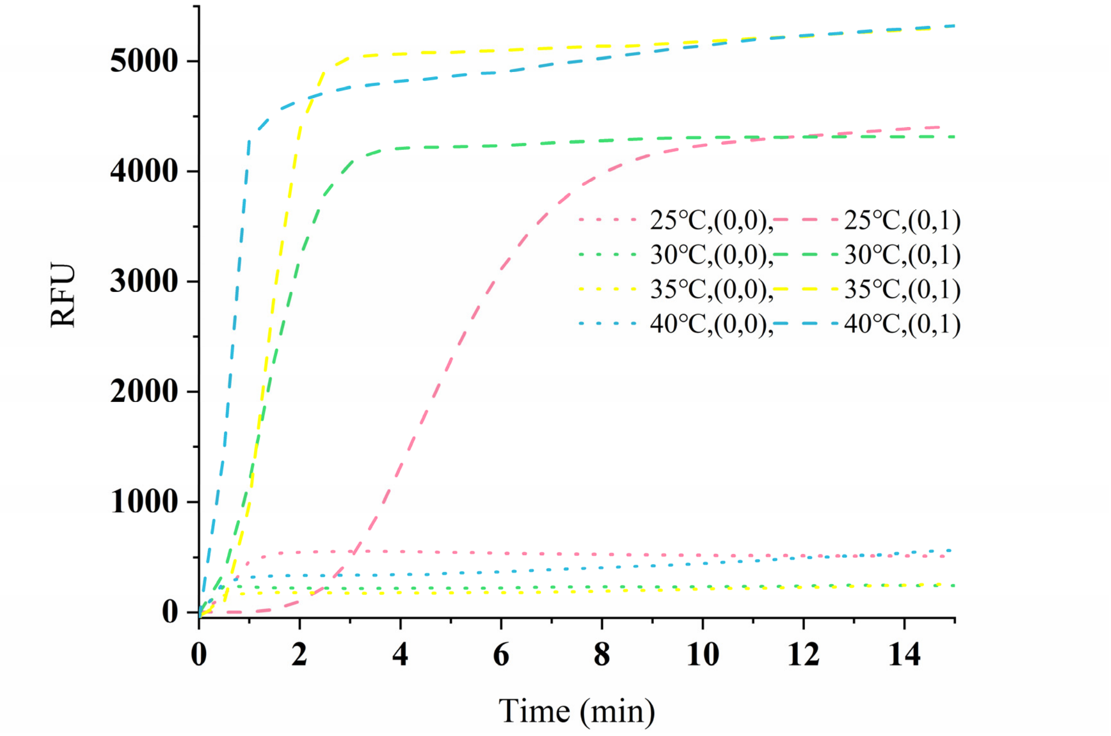

Supplementary Figure 6 Optimizations of the reaction temperature with 3.2 U Bst DNA polymerase and a single-rail OR gate. The reaction efficiency and leakage of the other gates or devices showed similar behaviors when we the reaction temperature was changed. Hence, we chose 35°C as our reaction temperature because satisfactory efficiency and leakage were observed at this temperature.

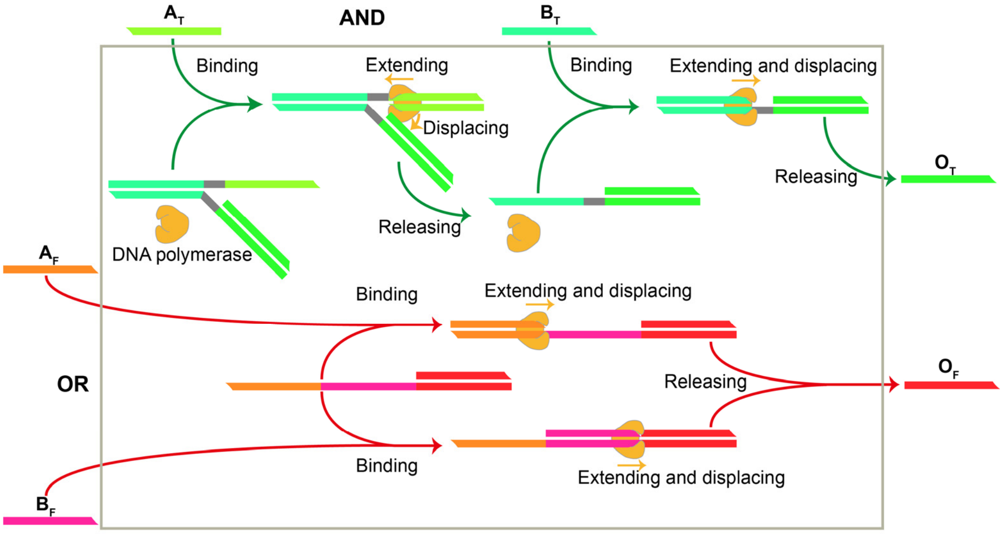

Supplementary Figure 7 Reaction details in dual-rail AND gate. The above diagram showed all possible reactions with all inputs. Only the downstream reactions of the given inputs would happen when a specific combination of inputs had been added to the system.

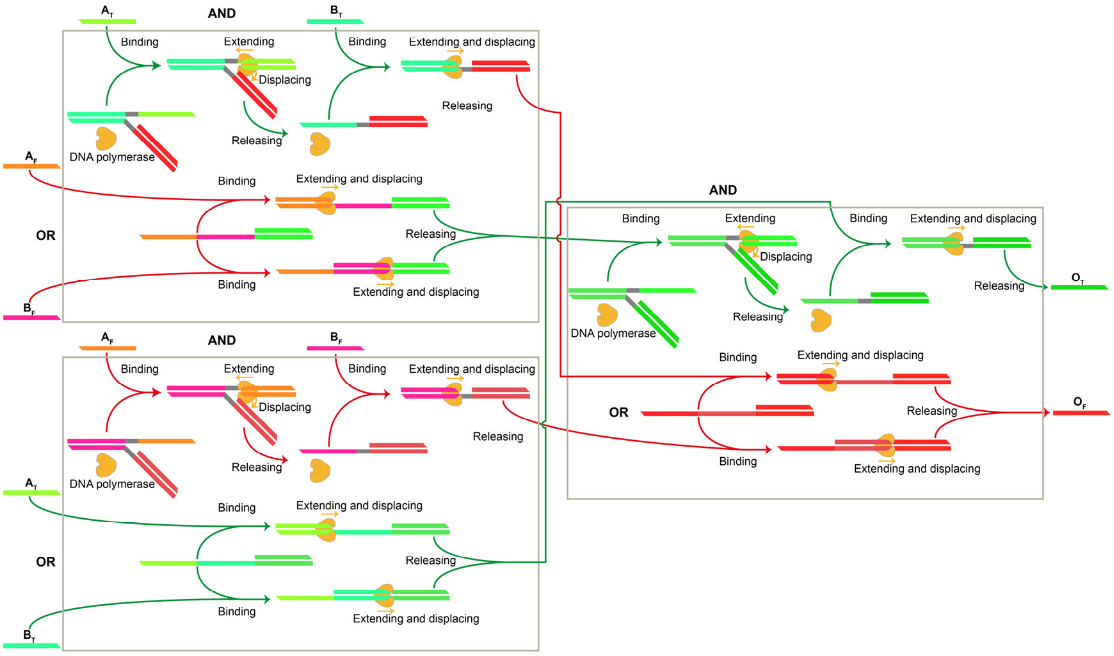

Supplementary Figure 8 Reaction details in dual-rail XOR gate. The above diagram showed all possible reactions with all inputs. Only the downstream reactions of the given inputs would happen when a specific combination of inputs had been added to the system.

a

Lane	1	2	3	4
Gate	AND			
A	0	0	1	1
B	0	1	0	1
Output	0	0	0	1

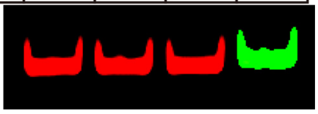

b

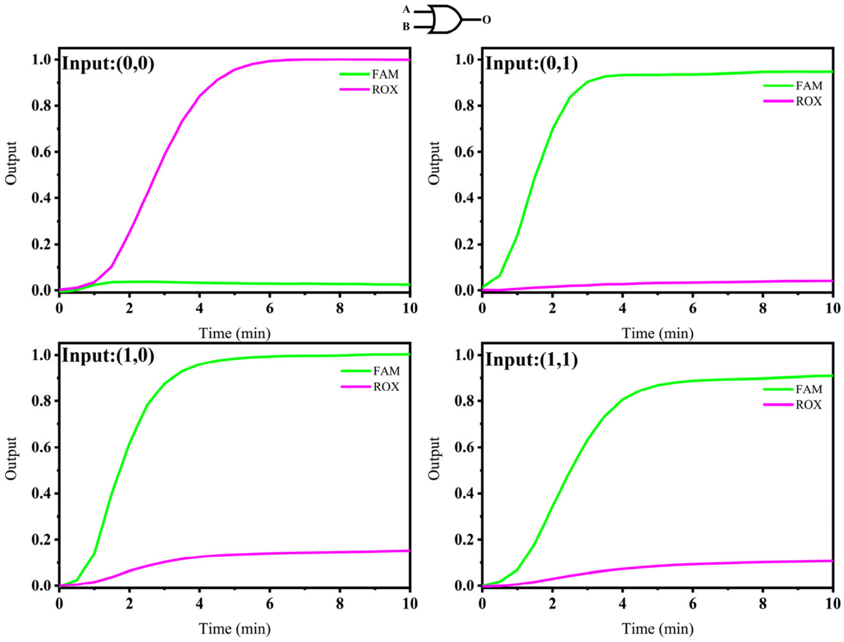

c

Lane	1	2	3	4
Gate	OR			
A	0	0	1	1
B	0	1	0	1
Output	0	1	1	1

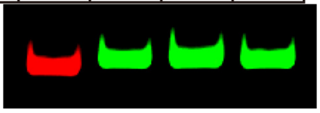

Supplementary Figure 9 PAGE analysis of the products produced by dual-rail AND **(a)** and OR **(c)** gates. Green and red were used to visualize the FAM and ROX channels, respectively. According to our definition, the green band and red band indicate TRUE and FALSE outputs, respectively. **b.** Reaction kinetics of the dual-rail OR gate with all possible combinations of inputs. The reaction was performed with 3.2 U Bst polymerase (large fragment), TRI and FRI at 35°C. The curve was plotted by transferring the cycle value into the reaction time. The outputs were normalized to the RFU values in the FAM and ROX channels with the highest signals. The FAM and ROX signals correspond to TRUE and FALSE returns, respectively. The sequences of the DNA strands are listed in the Supplementary Table 2. Source data are provided as a Source Data file.

a

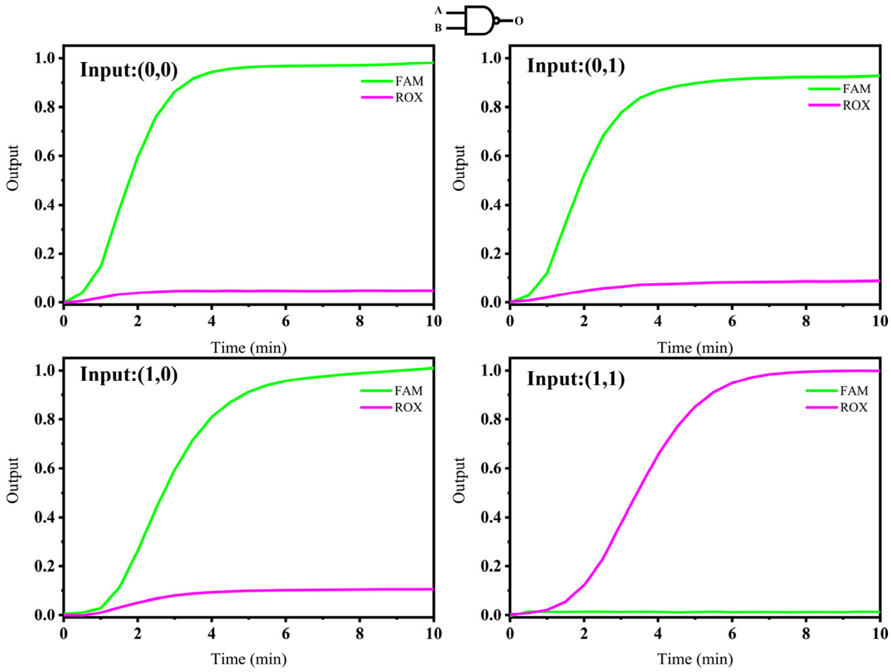

b

Lane	1	2	3	4
Gate	NAND			
A	0	0	1	1
B	0	1	0	1
Output	1	1	1	0

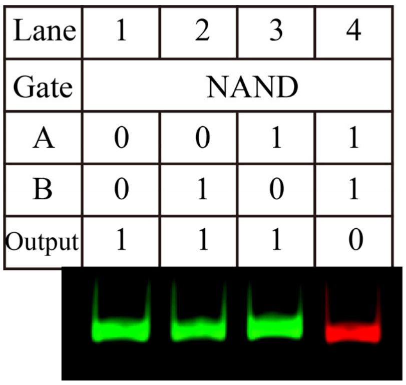

c

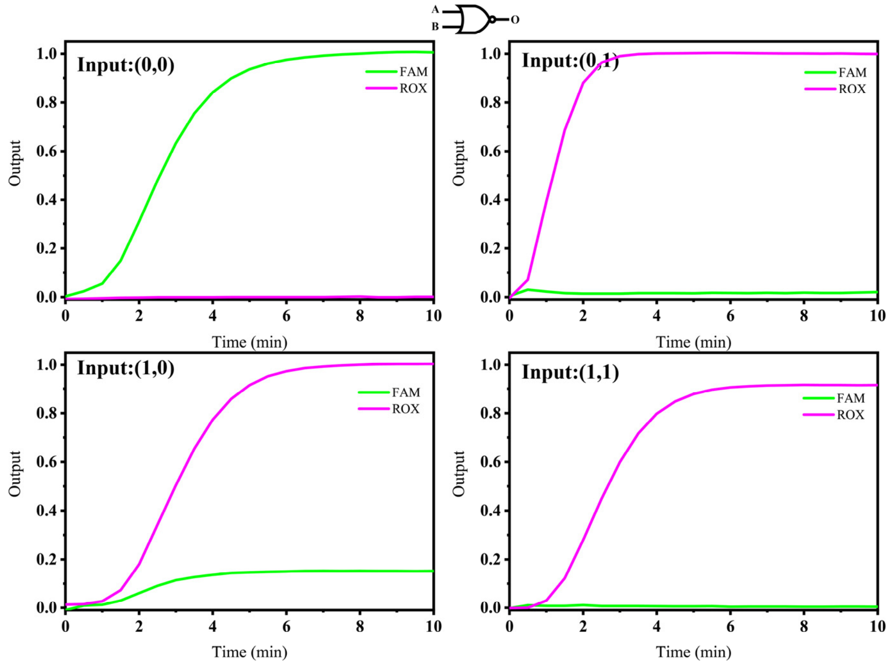

d

Lane	1	2	3	4
Gate	NOR			
A	0	0	1	1
B	0	1	0	1
Output	1	0	0	0

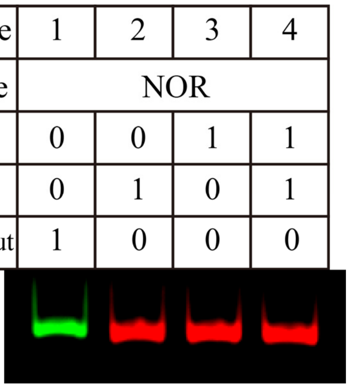

Supplementary Figure 10 Reaction kinetics of the dual-rail NAND **(a)** and NOR **(c)** gates with all possible combinations of inputs. The reaction was performed with 3.2 U Bst polymerase (large fragment), TRI and FRI at 35°C. The curve was plotted by transferring the cycle value into the reaction time. The outputs were normalized to the RFU values in the FAM and ROX channels with the highest signals. The FAM and ROX signals correspond to TRUE and FALSE returns, respectively. The sequences of the DNA strands are listed in the Supplementary Table 2. PAGE analysis of the products produced by the dual-rail NAND **(b)** and NOR **(d)** gates. Green and red were used to visualize the FAM and ROX channels, respectively. According to our definition, the green band and red band indicate TRUE and FALSE outputs, respectively. Source data are provided as a Source Data file.

a

Lane	1	2	3	4
Gate	XOR			
A	0	0	1	1
B	0	1	0	1
Output	0	1	1	0

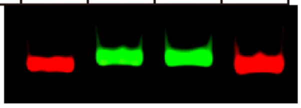

b

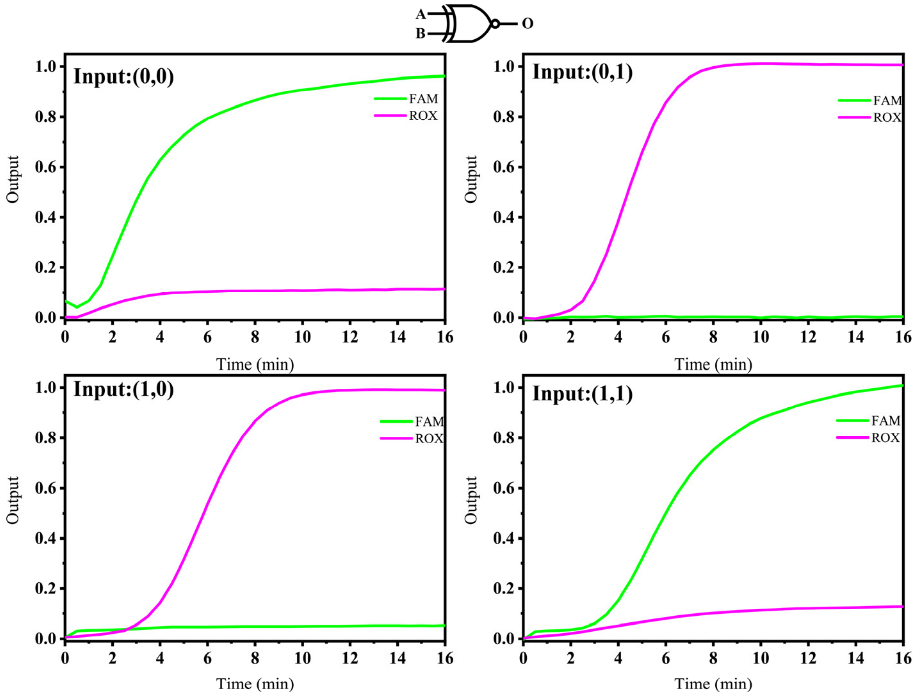

c

Lane	1	2	3	4
Gate	XNOR			
A	0	0	1	1
B	0	1	0	1
Output	1	0	0	1

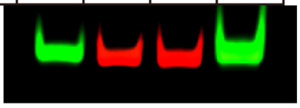

Supplementary Figure 11 PAGE analysis of the products produced by dual-rail XOR **(a)** and XNOR **(c)** gates. Green and red were used to visualize the FAM and ROX channels, respectively. According to our definition, the green band and red band indicate TRUE and FALSE outputs, respectively. **(b)** Reaction kinetics of the dual-rail XNOR gate with all possible combinations of inputs. The reaction was performed with 3.2 U Bst polymerase (large fragment), TRI and FRI at 35°C. The curve was plotted by transferring the cycle value into the reaction time. The outputs were normalized to the RFU values in the FAM and ROX channels with the highest signals. The FAM and ROX signals correspond to TRUE and FALSE returns, respectively. The sequences of the DNA strands are listed in the Supplementary Table 2. Source data are provided as a Source Data file.

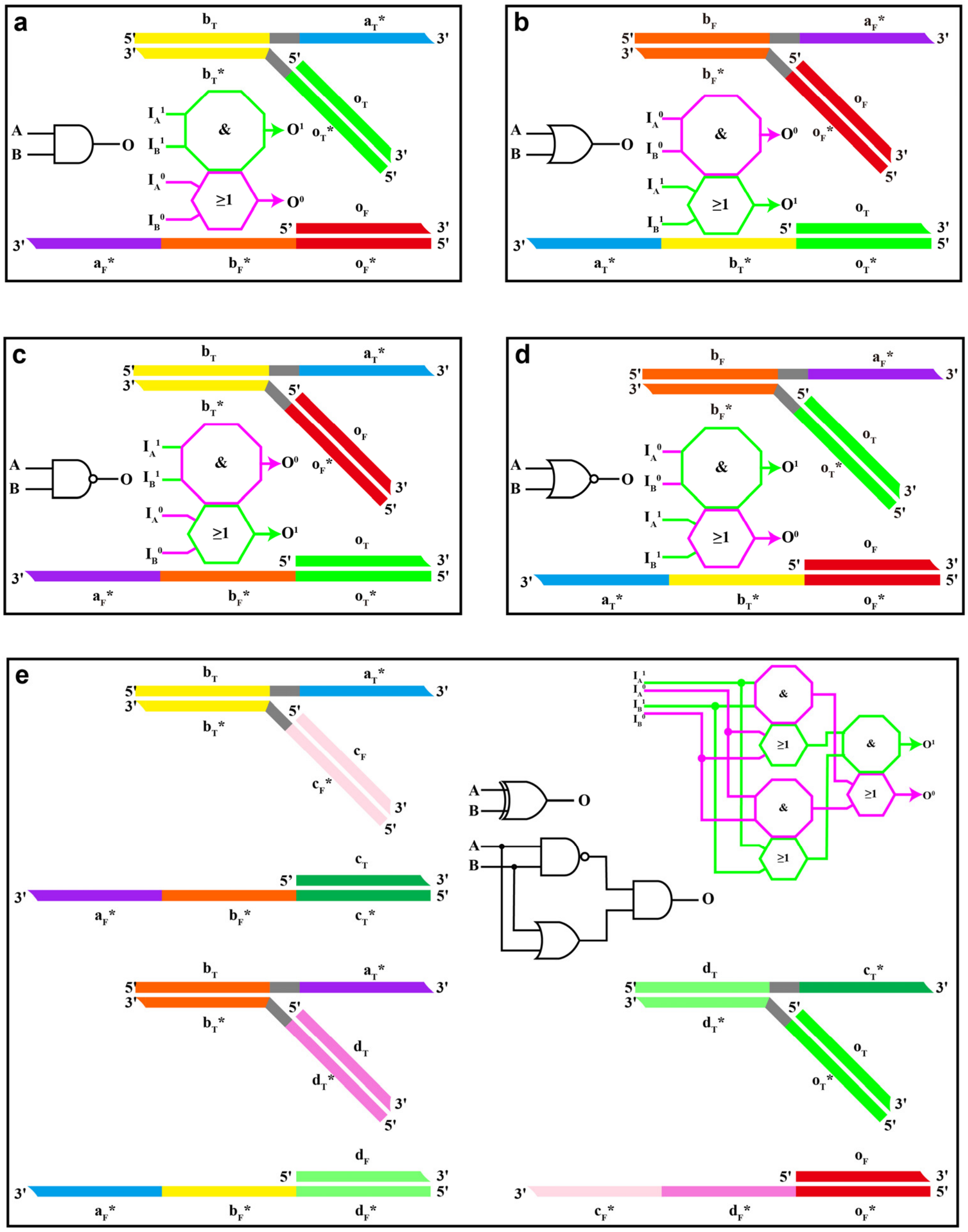

a
b
c
d
e
A
B
O
&
≥1
5'
3'
b_T
a_T^*
b_T^*
o_T
o_T^*
o_F
o_F^*
a_F^*
b_F^*
b_F
a_F
c_F
c_F^*
c_T
c_T^*
d_T
d_T^*
d_F
d_F^*
a_T^*
I_A^1
I_B^1
I_A^0
I_B^0
O^1
O^0

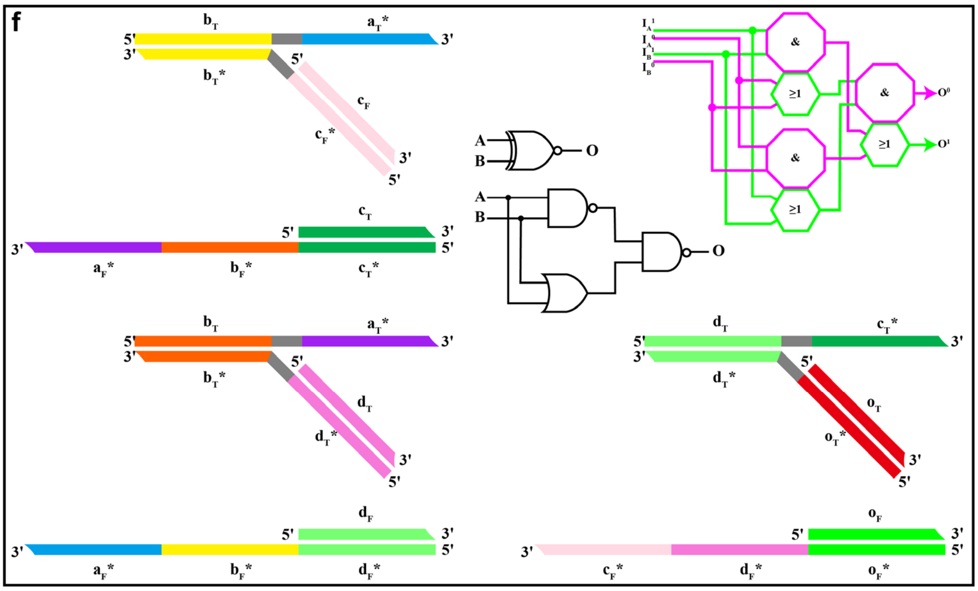

Supplementary Figure 12 Constructing basic dual-rail logic gates with the same inputs and outputs by rearranging the sequence of elements or adding additional sequence elements and DNA components. The lowercase letters indicate the sequence element, and * indicates complementary sequences. Each sequence element contains 18 bases. **(a-f)**: AND, OR, NAND, NOR, XOR and XNOR gates.

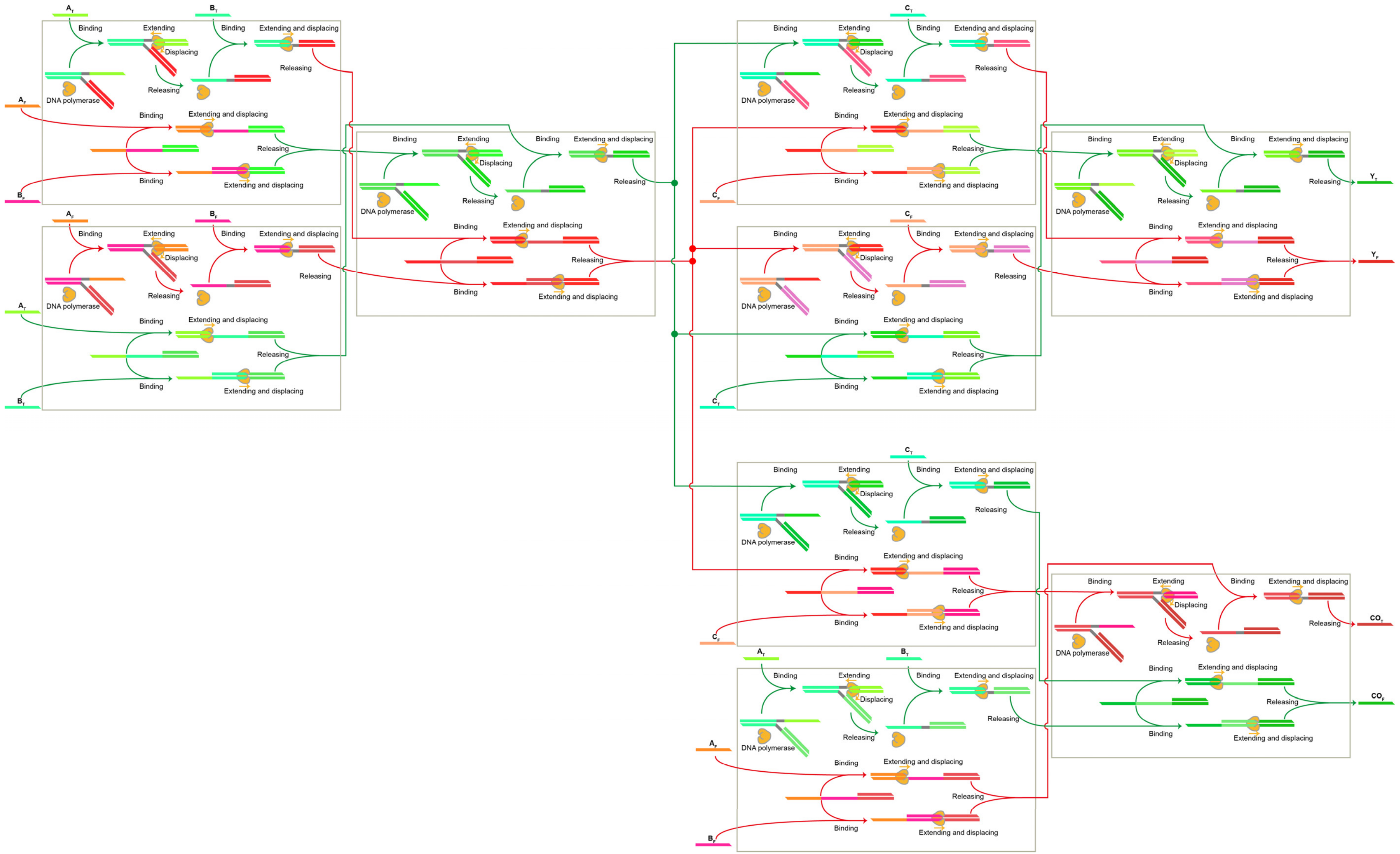

A_T
B_T
A_F
B_F
Binding
Extending
Displacing
Releasing
DNA polymerase
Extending and displacing
C_T
C_F
Y_T
Y_F
CO_T
CO_F

Supplementary Figure 13 Reaction details in the full adder. The above diagram showed all possible reactions with all inputs. Only the downstream reactions of the given inputs would happen when a specific combination of inputs had been added to the system.

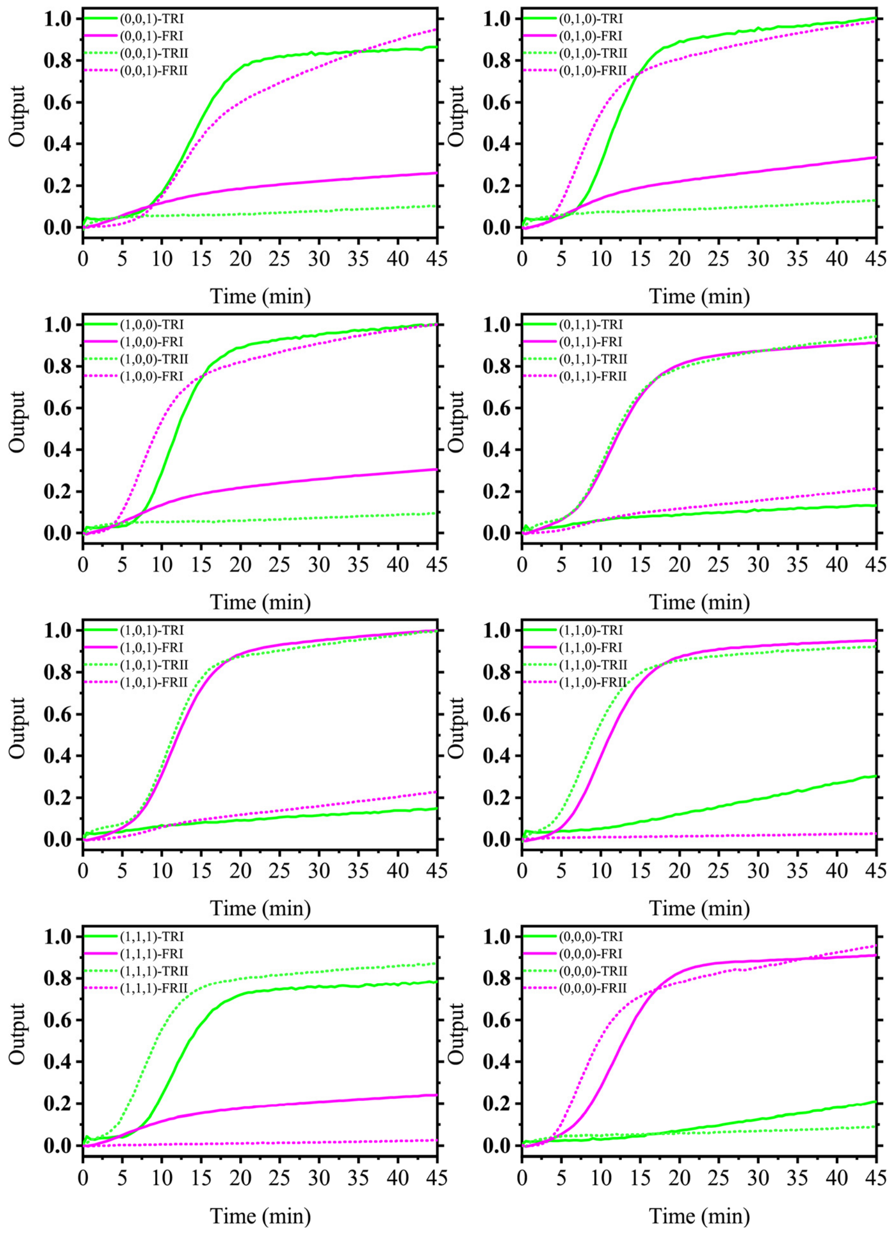

Supplementary Figure 14 Reaction kinetics of the full adder with all possible combinations of inputs. The reaction was performed with 12 U *Bst* polymerase (large fragment), TRI, FRI, TRII and FRII. The curve was plotted by transferring the cycle value into the reaction time. The outputs were normalized to the RFU values in the FAM, ROX, HEX and Cy5 channels with the highest signals. The TRI (FAM) and FRI (ROX) signals correspond to the TRUE and FALSE returns of S, respectively. The TRII (HEX) and FRII (Cy5) signals correspond to the TRUE and FALSE returns of C_{out}, respectively. The sequences of the DNA strands are listed in the Supplementary Table 2.

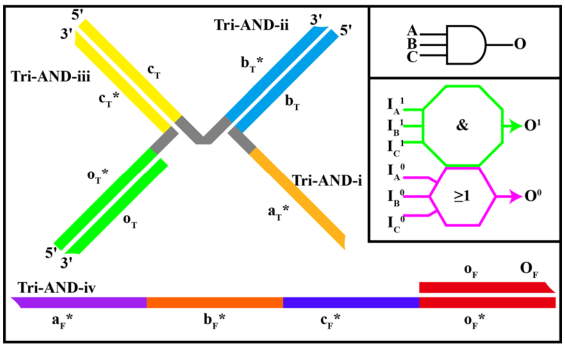

Supplementary Figure 15 Converting the 2-input dual-rail AND gate into a 3-input dual-rail AND gate. The names in capital letters are the sequence names, the names in lowercase letters are the sequence elements and * stands for complementary sequences. Each sequence element contains 18 bases. The dark spacer sequences in Tri-AND-i and Tri-AND-iii contain 4 bases, and the spacer sequence in Tri-AND-iii contains 8 bases.

lane	1	2	3	4	5	6	7	8	9	10
Tri-AND-I	+				+	+	+			
Tri-AND-II		+			+	+	+	+	+	
Tri-AND-III			+			+	+	+	+	+
OP_T				+			+		+	+

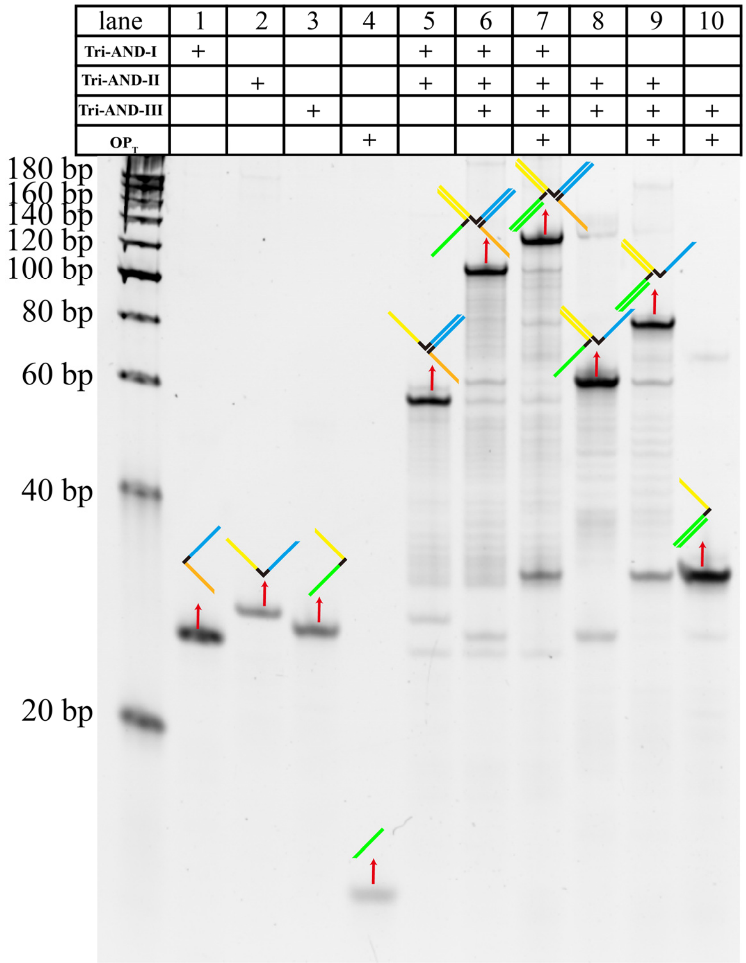

Supplementary Figure 16 PAGE results showed that the designed 3-input AND gate was assembled properly. Source data are provided as a Source Data file.

a

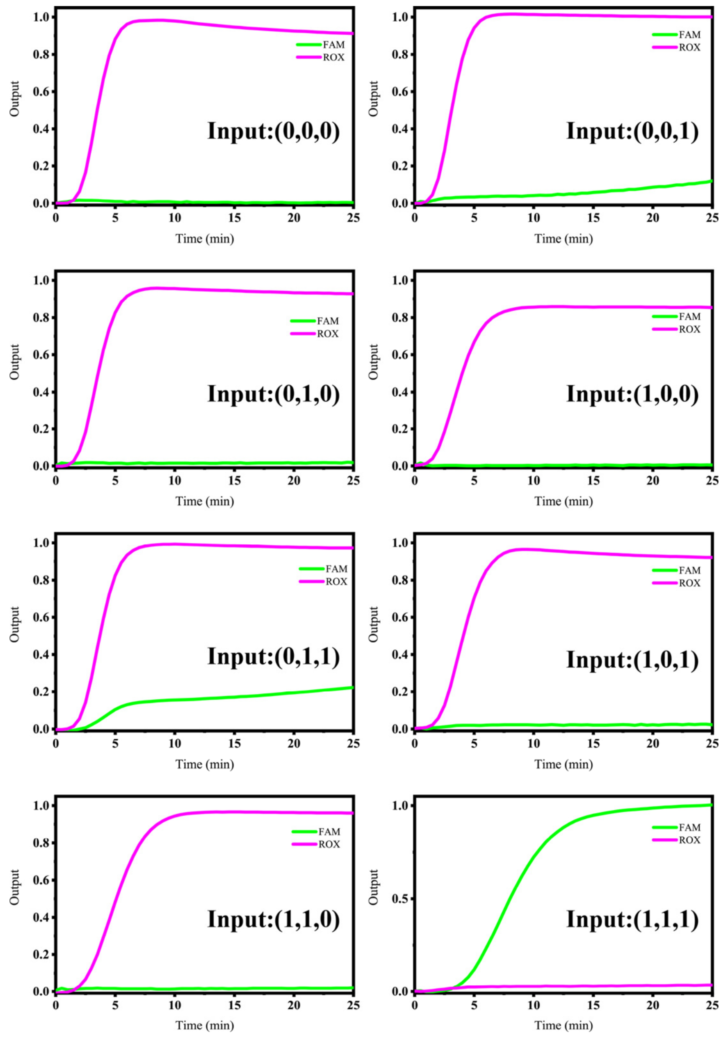

b

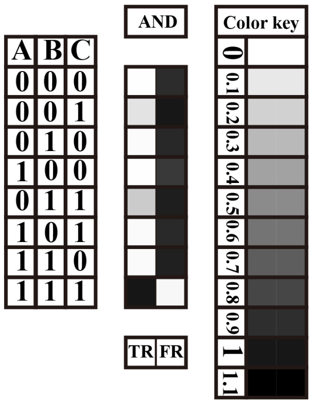

Supplementary Figure 17 (**a**) Reaction kinetics of the 3-input AND with all possible combinations of inputs. The reaction was performed with 3.2 U Bst polymerase (large fragment), TRI and FRI at 35°C. The curve was plotted by transferring the cycle value into the reaction time. The outputs were normalized to the RFU values in the FAM and ROX channels with the highest signals. The FAM and ROX signals correspond to TRUE and FALSE returns, respectively. The sequences of the DNA strands are listed in the Supplementary Table 2. (**b**) Summary of all the outputs from the Tri-AND gates.

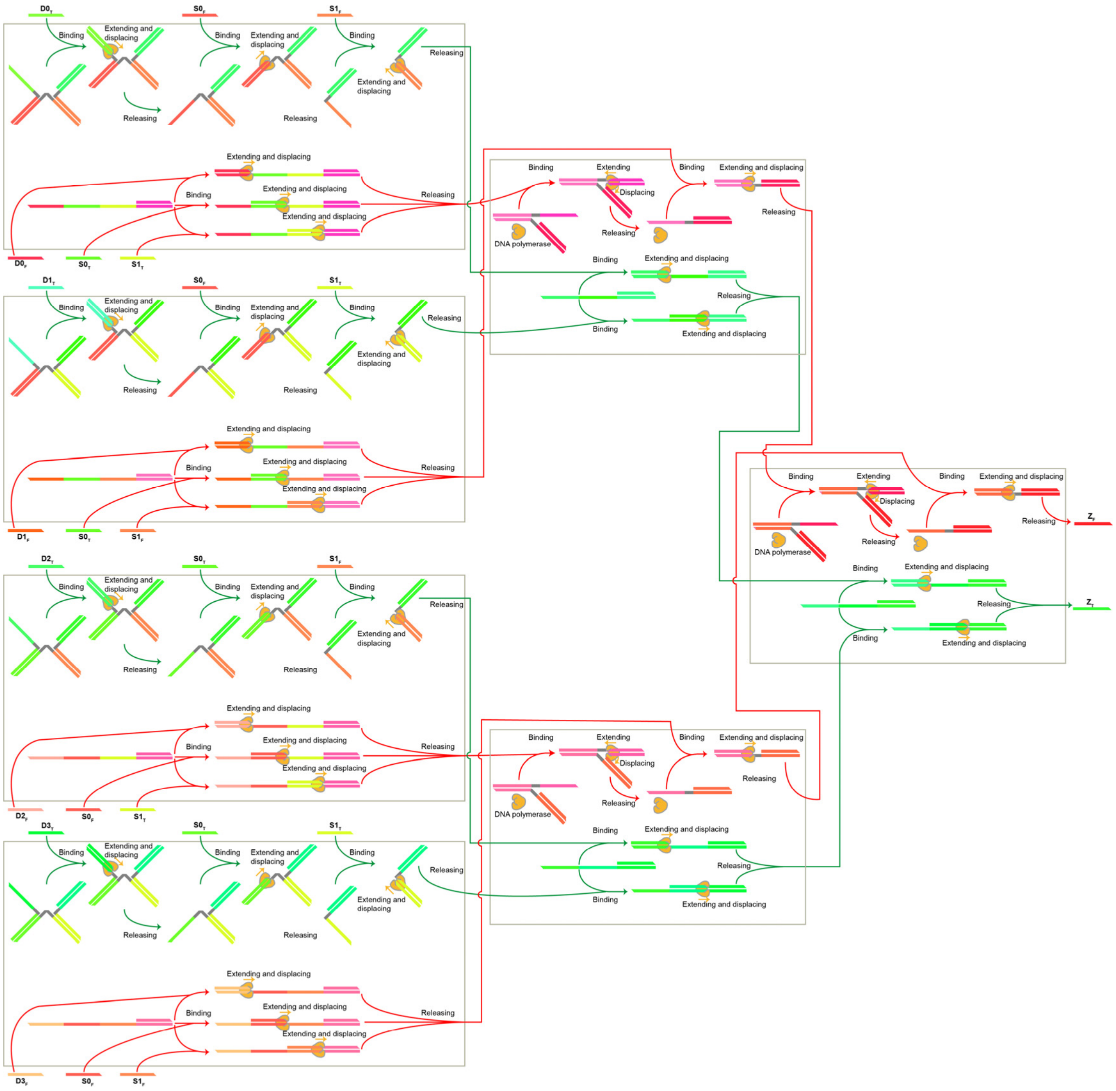

Supplementary Figure 18 Reaction details in the multiplexer. The above diagram showed all possible reactions with all inputs. Only the downstream reactions of the given inputs would happen when a specific combination of inputs had been added to the system.

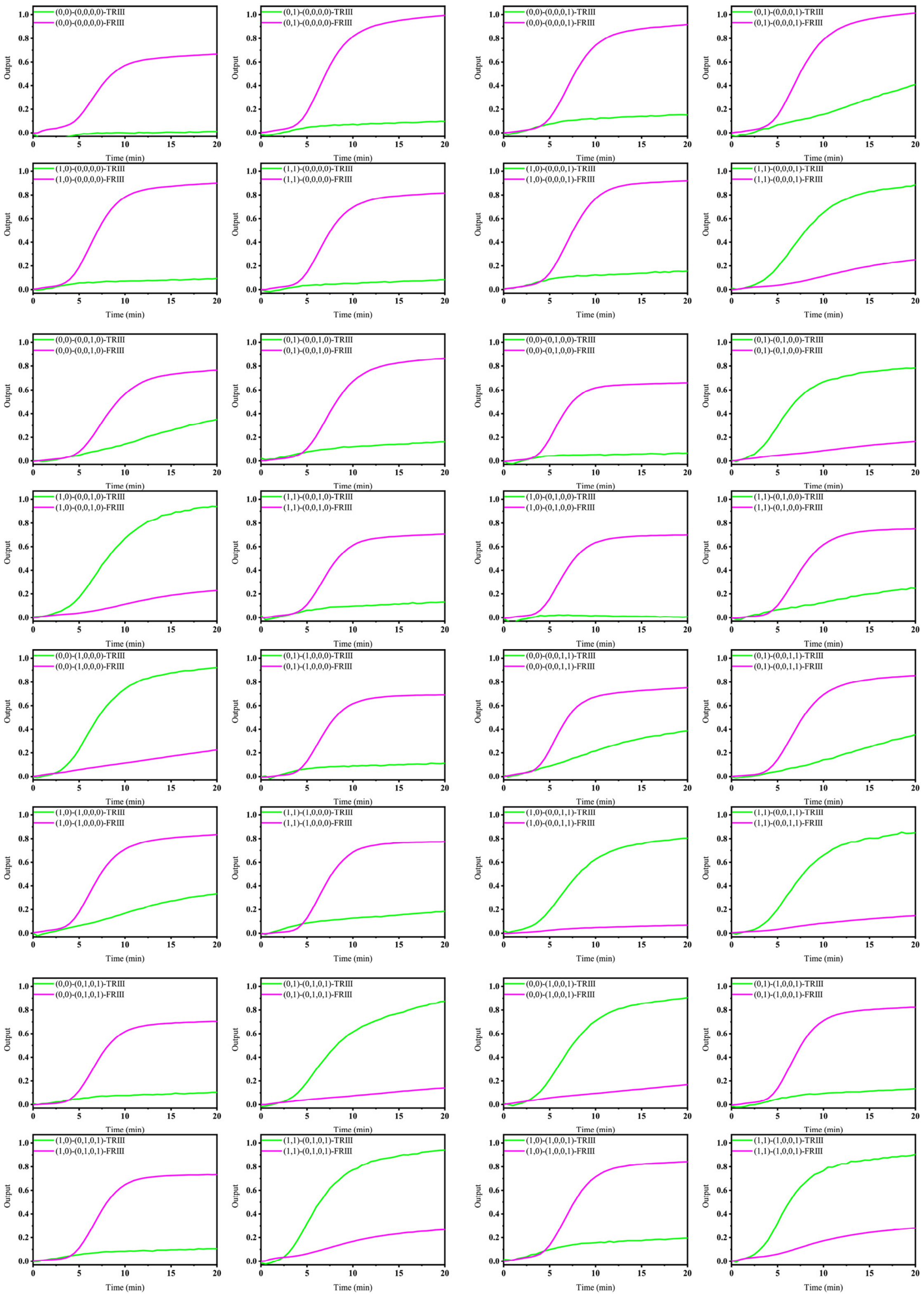
(0,0)-(0,0,0,0)-TRIII
(0,0)-(0,0,0,0)-FRIII
(0,1)-(0,0,0,0)-TRIII
(0,1)-(0,0,0,0)-FRIII
(0,0)-(0,0,0,1)-TRIII
(0,0)-(0,0,0,1)-FRIII
(0,1)-(0,0,0,1)-TRIII
(0,1)-(0,0,0,1)-FRIII
(1,0)-(0,0,0,0)-TRIII
(1,0)-(0,0,0,0)-FRIII
(1,1)-(0,0,0,0)-TRIII
(1,1)-(0,0,0,0)-FRIII
(1,0)-(0,0,0,1)-TRIII
(1,0)-(0,0,0,1)-FRIII
(1,1)-(0,0,0,1)-TRIII
(1,1)-(0,0,0,1)-FRIII
(0,0)-(0,0,1,0)-TRIII
(0,0)-(0,0,1,0)-FRIII
(0,1)-(0,0,1,0)-TRIII
(0,1)-(0,0,1,0)-FRIII
(0,0)-(0,1,0,0)-TRIII
(0,0)-(0,1,0,0)-FRIII
(0,1)-(0,1,0,0)-TRIII
(0,1)-(0,1,0,0)-FRIII
(1,0)-(0,0,1,0)-TRIII
(1,0)-(0,0,1,0)-FRIII
(1,1)-(0,0,1,0)-TRIII
(1,1)-(0,0,1,0)-FRIII
(1,0)-(0,1,0,0)-TRIII
(1,0)-(0,1,0,0)-FRIII
(1,1)-(0,1,0,0)-TRIII
(1,1)-(0,1,0,0)-FRIII
(0,0)-(1,0,0,0)-TRIII
(0,0)-(1,0,0,0)-FRIII
(0,1)-(1,0,0,0)-TRIII
(0,1)-(1,0,0,0)-FRIII
(0,0)-(0,0,1,1)-TRIII
(0,0)-(0,0,1,1)-FRIII
(0,1)-(0,0,1,1)-TRIII
(0,1)-(0,0,1,1)-FRIII
(1,0)-(1,0,0,0)-TRIII
(1,0)-(1,0,0,0)-FRIII
(1,1)-(1,0,0,0)-TRIII
(1,1)-(1,0,0,0)-FRIII
(1,0)-(0,0,1,1)-TRIII
(1,0)-(0,0,1,1)-FRIII
(1,1)-(0,0,1,1)-TRIII
(1,1)-(0,0,1,1)-FRIII
(0,0)-(0,1,0,1)-TRIII
(0,0)-(0,1,0,1)-FRIII
(0,1)-(0,1,0,1)-TRIII
(0,1)-(0,1,0,1)-FRIII
(0,0)-(1,0,0,1)-TRIII
(0,0)-(1,0,0,1)-FRIII
(0,1)-(1,0,0,1)-TRIII
(0,1)-(1,0,0,1)-FRIII
(1,0)-(0,1,0,1)-TRIII
(1,0)-(0,1,0,1)-FRIII
(1,1)-(0,1,0,1)-TRIII
(1,1)-(0,1,0,1)-FRIII
(1,0)-(1,0,0,1)-TRIII
(1,0)-(1,0,0,1)-FRIII
(1,1)-(1,0,0,1)-TRIII
(1,1)-(1,0,0,1)-FRIII
Output
Time (min)

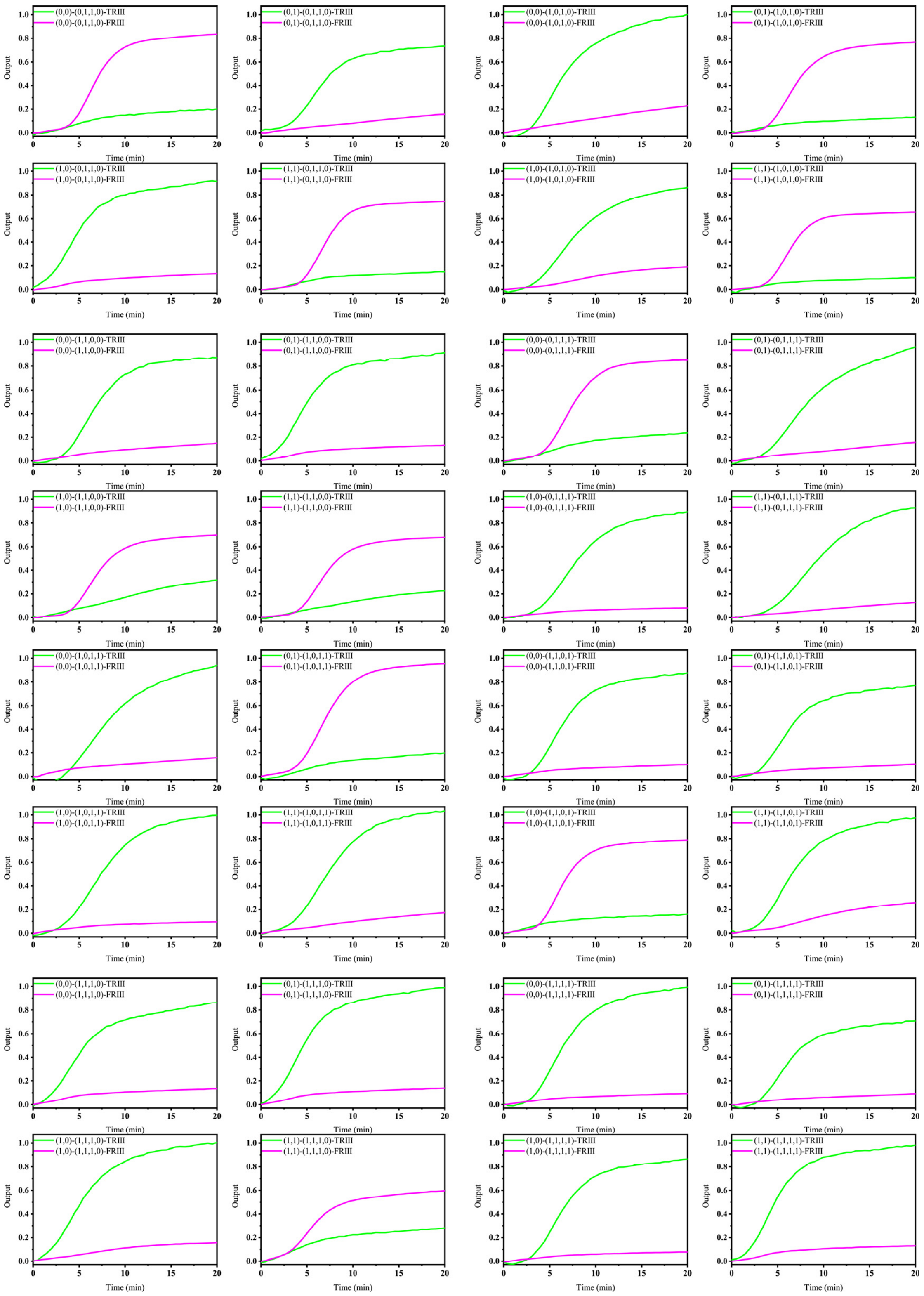

Supplementary Figure 19 Reaction kinetics of the multiplexer with all possible combinations of inputs. The reaction was performed with 6.4 U Bst polymerase (large fragment), TRIII and FRII. The curve was plotted by transferring the cycle value into the reaction time. The outputs were normalized to the RFU values in the FAM and ROX channels with the highest signals. The TRIII (FAM) and FRIII (ROX) signals correspond to TRUE and FALSE returns, respectively. The sequences of the DNA strands are listed in the Supplementary Table 2.

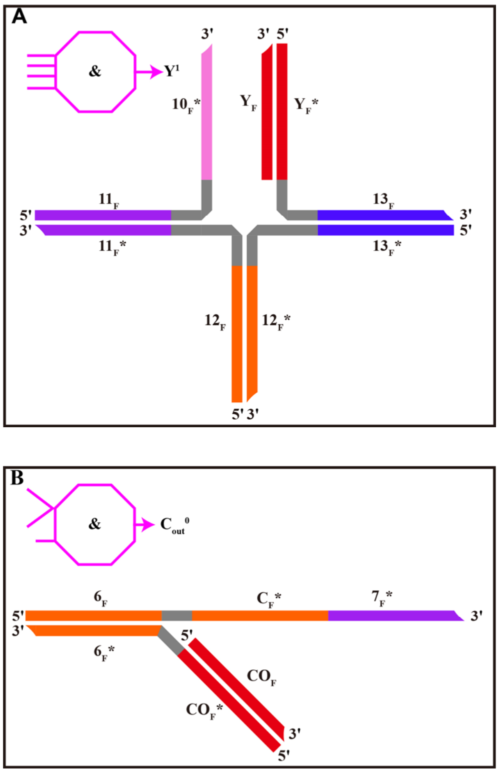

Supplementary Figure 20 Detail structures of the integrated logic gates shown in Figure 4d.

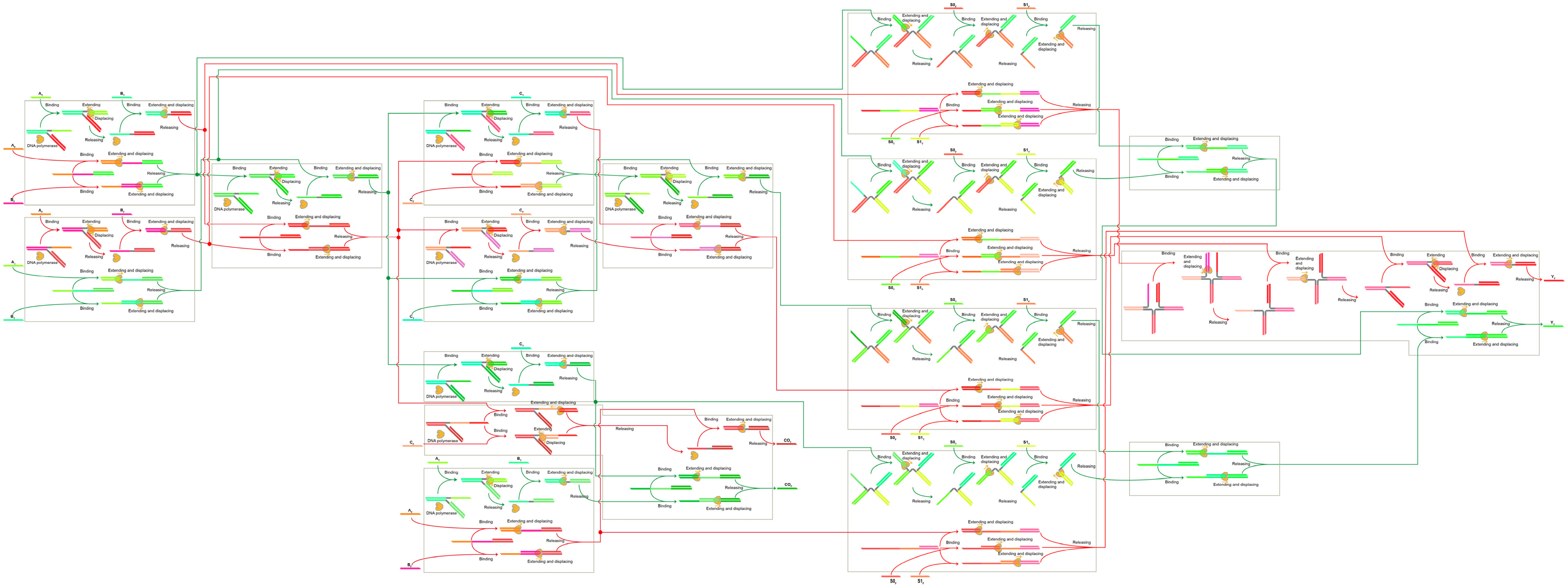

Supplementary Figure 21 Reaction details in the ALU. The above diagram showed all possible reactions with all inputs. Only the downstream reactions of the given inputs would happen when a specific combination of inputs had been added to the system.

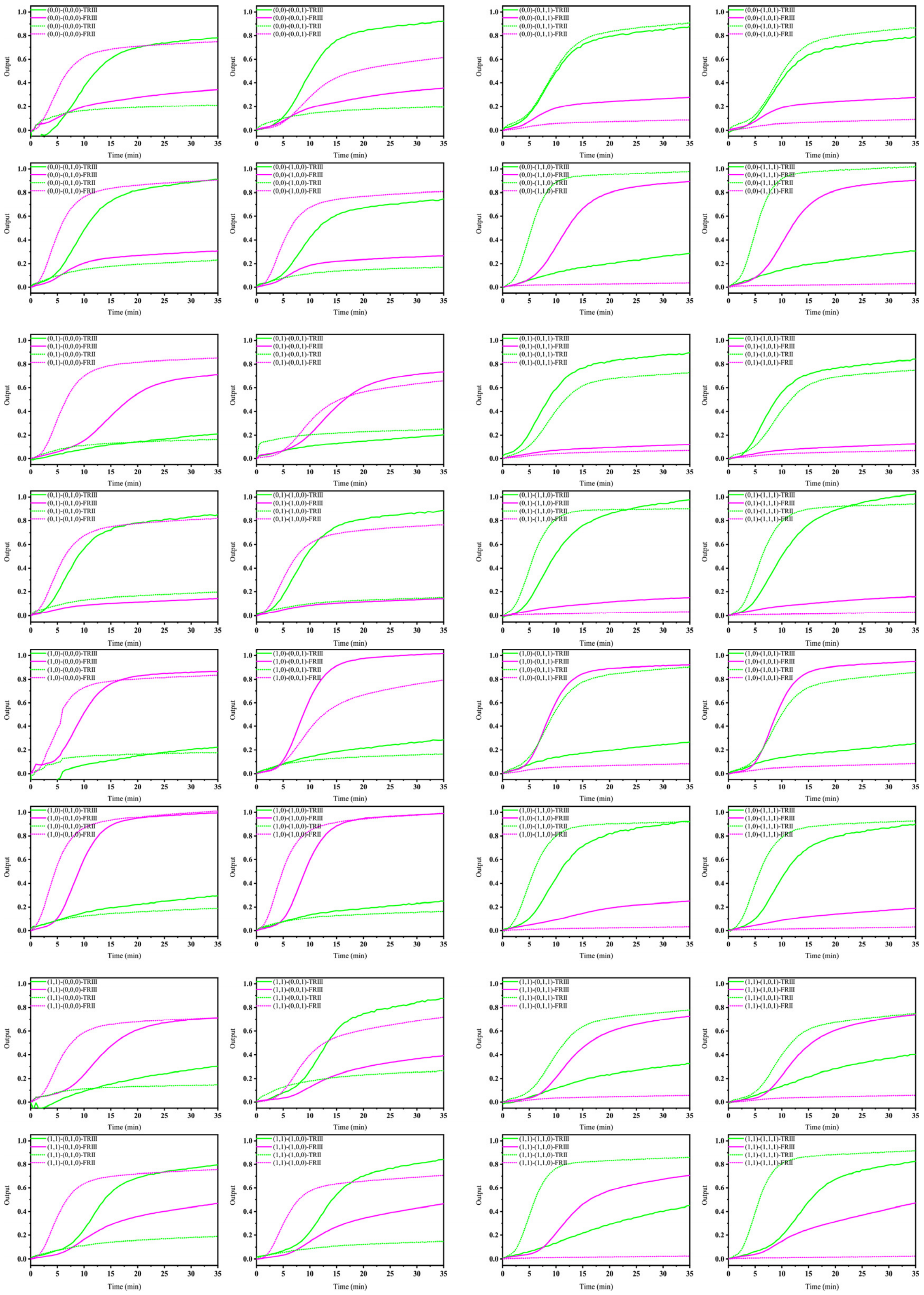

Supplementary Figure 22 Reaction kinetics of the ALU with all possible combinations of inputs. The reaction was performed with 12 U *Bst* polymerase (large fragment), TRIII, FRIII, TRII and FRII. The curve was plotted by transferring the cycle value into the reaction time. The outputs were normalized to the RFU values in the FAM, ROX, HEX and Cy5 channels with the highest signals. The TRIII (FAM) and FRIII (ROX) signals correspond to the TRUE and FALSE returns of Y, respectively. The TRII (HEX) and FRII (Cy5) signals correspond to the TRUE and FALSE returns of C_{out}, respectively. The sequences of the DNA strands are listed in the Supplementary Table 2.

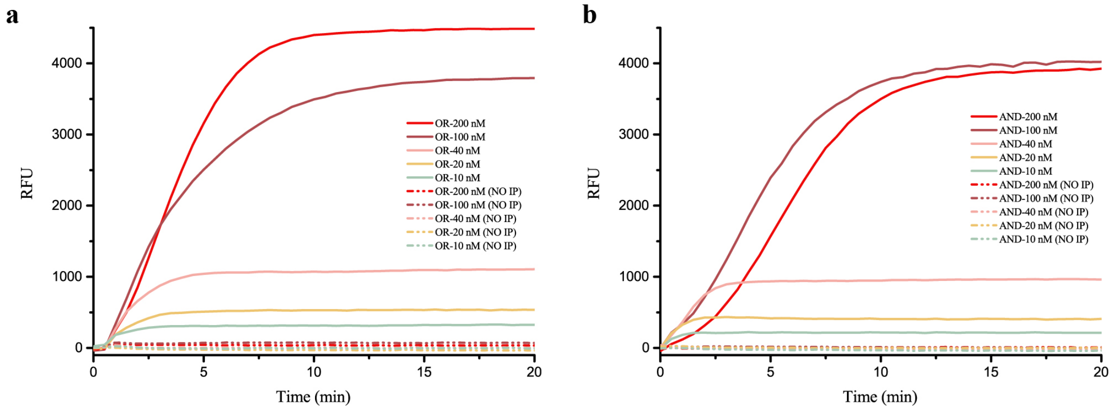

Supplementary Figure 23 Reaction kinetics of the single-rail OR (a) and AND (b) gates at various concentrations. The concentrations of input strands and logic gates are indicated in figure. The reaction was performed with 3.2 U Bst polymerase (large fragment) and TRI at 35°C. The curve was plotted by transferring the cycle value into the reaction time. It showed that our gates can work with high-efficiency at various concentrations.

Lane	1	2	3	4	5	6	7	8	9	10	11	12
**Group**	**1**		**2**		**3**		**4**		**5**		**6**	
**Gate**	**AND**	**OR**	**AND**	**OR**	**AND**	**OR**	**AND**	**OR**	**AND**	**OR**	**AND**	**OR**
**Buffer**	**TAE**		**TBE**		**TBE**		**TBE**		**TBE**		**TBE**	
**Mg^{2+}**	**12.5 mM**		**12.5 mM**		**6.25 mM**		**-**		**-**		**-**	
**Temperature**	**RT**		**RT**		**RT**		**4°C**		**RT**		**RT**	
**Purity of oligos**	**RPC**		**RPC**		**RPC**		**RPC**		**RPC**		**PAGE**	

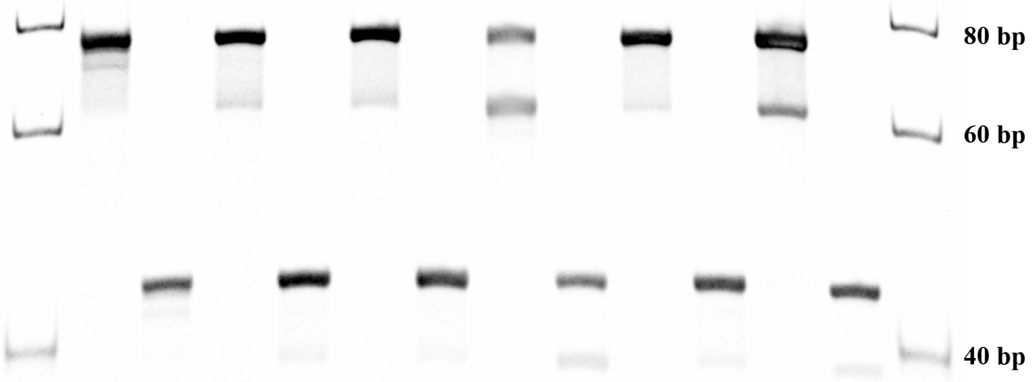

Supplementary Figure 24 PAGE analysis of different purified DNA components. The present of Mg^{2+} induced some undesired band between 40-60bp. Recover at low temperature lead significant impurity. Source data are provided as a Source Data file.

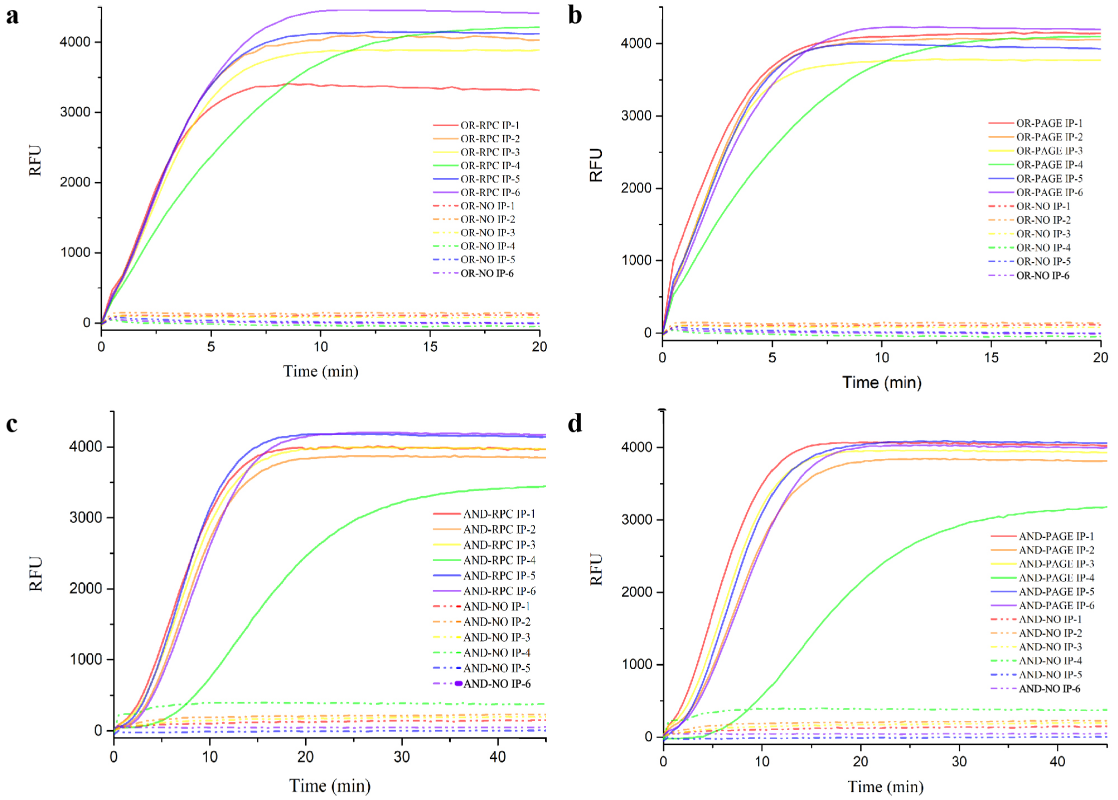

Supplementary Figure 25 The performance of different purified DNA components and inputs. The number 1-6 corresponding the group number in Supplementary Figure 24. Recover with TBE buffer at room temperature had the lowest leakage in general. As the similar performance of RPC and PAGE purified strands to construct the gates and the huge amount of demand, we chosen cheaper RPC purified strands. In contrast, we chosen PAGE purified inputs for their high purity.

Supplementary Table 1 Comparison with other DNA computational systems.

	Our work	Strands displacement based on see-saw gates[1]	Simply strands displacement[2]	Compact DNA strand displacement circuits[3]
Experiments with real DNA	Yes	Yes	Yes	No
Computation type	Boolean and arithmetic logic	Boolean and arithmetic logic	Boolean and arithmetic logic	Analog computations
Computation time	5- 40 minutes	4-12 hours	12 hours	~ 27.7 hours
Cascade circuit	Available	Available	Available	Available
Functional complete set	Available	Available	Not available	Not available
Strands/components for single-rail AND gate	3/1	7/4	11/6	-
Strands/components for single-rail OR gate	2/1	7/4	No construction	-
Strands/components for dual rail AND	5/2	14/8	No construction	-
Strands/components for half adder	20/8	56/32(count with typical construction)	20/11(single-rail)	-
Strategy for integrating gates	Offered	Not offered	Not offered	Not offered
Representative devices	Full adder, MUX 4:1 and ALU	A circuit computed square-root	Half adder	DNA circuits to compute functions sqrt(x), ln(x) and exp(x)

Supplementary Table 2 Sequence list of logic gates and circuits.

Application	Name	Sequence (5'-3')
Input for logic gates	A_T	TGTATGTTTATGCGTTTT
	B_T	TTGAGAGGAGGAGTCGTC
	A_F	CTTGACCACTCTAGCAGT
	B_F	ATCGAACTAGTCACATAA
Single-rail AND gate (top 3 strands) and dual-rail AND gate	AND-i	TTGAGAGGAGGAGTCGTCTACCAAAACGCATAAACATACA
	AND-ii	TTACCTGAGACTACTTGGACTGGACGACTCCTCCTCTCAA
	OP_T	CCAAGTAGTCTCAGGTAA
	AND-iii	CTTCATACCCGTGCTGGCTTATGTGACTAGTTCGATCCGGCT ATAGTGGTCAAG
	OP_F	GCCAGCACGGGTATGAAG
Single-rail OR gate (top 3 strands) and dual-rail OR gate	OR-i	ATCGAACTAGTCACATAACTGGCCGGCTATAGTGGTCAAG
	OR-ii	CTTCATACCCGTGCTGGCTTCATTATGTGACTAGTTCGAT
	OP_F	GCCAGCACGGGTATGAAG
	OR-ii	TTACCTGAGACTACTTGGGACGACTCCTCCTCTCAAAAAACG CATAAACATACA
	OP_T	CCAAGTAGTCTCAGGTAA
Dual-rail NAND gate	NAND-i	TTGAGAGGAGGAGTCGTCTACCAAAACGCATAAACATACA
	NAND-ii	CTTCATACCCGTGCTGGCACTGGACGACTCCTCCTCTCAA
	OP_F	GCCAGCACGGGTATGAAG
	NAND-iii	TTACCTGAGACTACTTGGTTATGTGACTAGTTCGATCCGGCT ATAGTGGTCAAG
	OP_T	CCAAGTAGTCTCAGGTAA
Dual-rail NOR gate	NOR-i	ATCGAACTAGTCACATAACTGGCCGGCTATAGTGGTCAAG
	NOR-ii	TTACCTGAGACTACTTGGTTCATTATGTGACTAGTTCGAT
	OP_T	CCAAGTAGTCTCAGGTAA
	NOR-iii	CTTCATACCCGTGCTGGCGACGACTCCTCCTCTCAAAAAACG CATAAACATACA
	OP_F	GCCAGCACGGGTATGAAG
Dual-rail XOR gate	XOR(NAND)-i	TTGAGAGGAGGAGTCGTCTACCAAAACGCATAAACATACA
	XOR(NAND)-ii	ACGATTACACGGAGCCTAACTGGACGACTCCTCCTCTCAA
	OPF(XOR-i)	TAGGCTCCGTGTAATCGT
	XOR(NAND)-iii	GTAATCTCCAATCTATGCTTATGTGACTAGTTCGATCCGGCT ATAGTGGTCAAG
	OPT(XOR-i)	GCATAGATTGGAGATTAC
	XOR(OR)-i	ATCGAACTAGTCACATAACTGGCCGGCTATAGTGGTCAAG
	XOR(OR)-ii	CATAAATTCTGCCGGTGGTTCATTATGTGACTAGTTCGAT
	OPF(XOR-ii)	CCACCGGCAGAATTTATG

	XOR(OR)-iii	TACTTACTGTGTGACGCCGACGACTCCTCCTCTCAAAAAACGCATAAACATACA
	OPT(XOR-ii)	GGCGTCACACAGTAAGTA
	XOR(AND)-i	GGCGTCACACAGTAAGTATACCGTAATCTCCAATCTATGC
	XOR(AND)-ii	TTACCTGAGACTACTTGGACTGTACTTACTGTGTGACGCC
	OP_T	CCAAGTAGTCTCAGGTAA
	XOR(AND)-iii	CTTCATACCCGTGCTGGCACGATTACACGGAGCCTACATAAATTCTGCCGGTGG
	OP_F	GCCAGCACGGGTATGAAG
Dual-rail XNOR gate	XNOR(NAND)-i	TTGAGAGGAGGAGTCGTCTACCAAAACGCATAAACATACA
	XNOR(NAND)-ii	ACGATTACACGGAGCCTAACTGGACGACTCCTCCTCTCAA
	OPF(XNOR-i)	TAGGCTCCGTGTAATCGT
	XNOR(NAND)-iii	GTAATCTCCAATCTATGCTTATGTGACTAGTTCGATCCGGCTATAGTGGTCAAG
	OPT(XNOR-i)	GCATAGATTGGAGATTAC
	XNOR(OR)-i	ATCGAACTAGTCACATAACTGGCCGGCTATAGTGGTCAAG
	XNOR(OR)-ii	CATAAATTCTGCCGGTGGTTCATTATGTGACTAGTTCGAT
	OPF(XNOR-ii)	CCACCGGCAGAATTTATG
	XNOR(OR)-iii	TACTTACTGTGTGACGCCGACGACTCCTCCTCTCAAAAAACGCATAAACATACA
	OPT(XNOR-ii)	GGCGTCACACAGTAAGTA
	XNOR(NAND2)-i	GGCGTCACACAGTAAGTATACCGTAATCTCCAATCTATGC
	XNOR(NAND2)-ii	CTTCATACCCGTGCTGGCACTGTACTTACTGTGTGACGCC
	OP_F	GCCAGCACGGGTATGAAG
	XNOR(NAND2)-iii	TTACCTGAGACTACTTGGCATAAATTCTGCCGGTGGACGATTACACGGAGCCTA
	OP_T	CCAAGTAGTCTCAGGTAA
Dual-rail 3-input AND gate	Tri-AND-i	TGTATGTTTATGCGTTTTATGCAGCACCTTTCTCACGGGA
	Tri-AND-ii	TTGAGAGGAGGAGTCGTCTACGGGGCAAAACGCATAAACATACA
	Tri-AND-iii	TTACCTGAGACTACTTGGACTGGACGACTCCTCCTCTCAA
	OP_T	CCAAGTAGTCTCAGGTAA
	Tri-AND-iv	CTTCATACCCGTGCTGGCTTATGTGACTAGTTCGATCCGGCTATAGTGGTCAAGATCTCGAGGGACTTTGCG

	OP_F	GCCAGCACGGGTATGAAG
Inputs for full adder	A_T	AGGAACCGGCACAGAGAT
	B_T	CTTATATCCGCACTCTGG
	A_F	CAGTCAGTGGTATTGTCG
	B_F	AGGTCCTAGGCTAGCTGT
	C_T	ACCAACCGAATATAACGC
	C_F	AATTCTAAGCAGCCGTCA
Full adder	N2-i	CAGTCAGTGGTATTGTCGTCGAATCTCTGTGCCGGTTCCT
	N2-ii	ACCCATTAAGCCTAACTGATATCGACAATACCACTGACTG
	L8F	CAGTTAGGCTTAATGGGT
	N2-iii	TATGTGTTCGTGAGTACCCCAGAGTGCGGATATAAGACAGC TAGCCTAGGACCT
	L8T	GGTACTCACGAACACATA
	O6-i	AGGTCCTAGGCTAGCTGTAGTCCCAGAGTGCGGATATAAG
	O6-ii	TCCACTGAAGAACGACCTTGTGACAGCTAGCCTAGGACCT
	L9F	AGGTCGTTCTTCAGTGGA
	O6-iii	GATGGTCTTAGCTTGGTTATCTCTGTGCCGGTTCCTCGACAA TACCACTGACTG
	L9T	AACCAAGCTAAGACCATC
	A8-i	AACCAAGCTAAGACCATCGTTGTATGTGTTCGTGAGTACC
	A8-ii	GCATACTTGTGGGTCTTTGTTGGATGGTCTTAGCTTGGTT
	L7T	AAAGACCCACAAGTATGC
	A8-iii	TACTCTCACTCGATCACCACCCATTAAGCCTAACTGCCTCCA CTGAAGAACGACCT
	L7F	GGTGATCGAGTGAGAGTA
	N1-i	AAAGACCCACAAGTATGCTCTCGCGTTATATTCGGTTGGT
	N1-ii	TGTCTTCGTCCTAGTTCATTTAGCATACTTGTGGGTCTTT
	L3F	TGAACTAGGACGAAGACA
	N1-iii	TAACGAAGACCTGTGGATTGACGGCTGCTTAGAATTGTACTC TCACTCGATCACC
	L3T	ATCCACAGGTCTTCGTTA
	O5-i	GGTGATCGAGTGAGAGTATGGTTGACGGCTGCTTAGAATT
	O5-ii	TGTCCCTTGCAACAGTAAGTCCTACTCTCACTCGATCACC
	L4F	TTACTGTTGCAAGGGACA
	O5-iii	CGGACATCATGAGCTCTTCGCGTTATATTCGGTTGGTTGCAT ACTTGTGGGTCTTT
	L4T	AAGAGCTCATGATGTCCG
	A5-i	AAGAGCTCATGATGTCCGAAGATAACGAAGACCTGTGGAT
	A5-ii	TTACCTGAGACTACTTGGACGCCGGACATCATGAGCTCTT
	OPT	CCAAGTAGTCTCAGGTAA
	A5-iii	CTTCATACCCGTGCTGGCTGTCTTCGTCCTAGTTCATGTCCCT TGCAACAGTAA
	OPF	GCCAGCACGGGTATGAAG
	A6-i	AAAGACCCACAAGTATGCTCTCGCGTTATATTCGGTTGGT
	A6-ii	CTCATCACTCTGGGTCTGCTTCGCATACTTGTGGGTCTTT
	L5T	CAGACCCAGAGTGATGAG

	A6-iii	GGGTAGGTTGAGACTTTATGACGGCTGCTTAGAATTGTACTCTCACTCGATCACC
	L5F	TAAAGTCTCAACCTACCC
	A7-i	CAGTCAGTGGTATTGTCGTCCTATCTCTGTGCCGGTTCCT
	A7-ii	GACACTTCTCTCTAGCTTTCCTCGACAATACCACTGACTG
	L6T	AAGCTAGAGAGAAGTGTC
	A7-iii	ACGATTACATGCAGCCTACCAGAGTGCGGATATAAGACAGCTAGCCTAGGACCT
	L6F	TAGGCTGCATGTAATCGT
	O4-i	TAGGCTGCATGTAATCGTTGTTGGGTAGGTTGAGACTTTA
	O4-ii	ACTCACATTCCTGGAATCATTCACGATTACATGCAGCCTA
	COF	GATTCCAGGAATGTGAGT
	O4-iii	CGGTCCAATTAGCTCAAACCTCATCACTCTGGGTCTGGACACTTCTCTCTAGCTT
	COT	TTTGAGCTAATTGGACCG
Select codes for MUX 4:1	$S0_F$	CAATGTGTGACTCCAAGA
	$S0_T$	GTATTAGTCAGTTCTCCC
	$S1_F$	GATTCGTCGCAATTATCG
	$S1_T$	TCTACTCTATGGGCCTCT
Inputs for MUX 4:1	$D0_T$	GGTACTCACGAACACATA
	$D0_F$	CAGTTAGGCTTAATGGGT
	$D1_T$	AACCAAGCTAAGACCATC
	$D1_F$	AGGTCGTTCTTCAGTGGA
	$D2_T$	AAGCTAGAGAGAAGTGTC
	$D2_F$	TAGGCTGCATGTAATCGT
	$D3_T$	CCAAGTAGTCTCAGGTAA
	$D3_F$	GCCAGCACGGGTATGAAG
MUX 4:1	T1-i	TCTACTCTATGGGCCTCTTCTCGGGAGAACTGACTAATAC
	T1-ii	GGTACTCACGAACACATATTATTCTTAGAGGCCCATAGAGTAGA
	T1-iii	GATTTGCCGCTTAGTCTGCCTTTATGTGTTCGTGAGTACC
	L10T	CAGACTAAGCGGCAAATC
	T1-iv	CTGCATGCCCGTTAGAAATCTTGGAGTCACACATTGCGATAATTGCGACGAATCACCCATTAAGCCTAACTG
	L10F	TTTCTAACGGGCATGCAG
	T2-i	GATTCGTCGCAATTATCGGTGTGGGAGAACTGACTAATAC
	T2-ii	AACCAAGCTAAGACCATCTGTGTGTGCGATAATTGCGACGAATC
	T2-iii	AGGTGTAGGGTCTCGTATAGTCGATGGTCTTAGCTTGGTT
	L11T	ATACGAGACCCTACACCT
	T2-iv	ATCCTGGAGTAACTTACGTCTTGGAGTCACACATTGAGAGGCCCATAGAGTAGAAGTCCACTGAAGAACGACCT
	L11F	CGTAAGTTACTCCAGGAT
	T3-i	TCTACTCTATGGGCCTCTTTAATCTTGGAGTCACACATTG

	T3-ii	AAGCTAGAGAGAAGTGTCGGTTGGTTAGAGGCCCATAGAGTAGA
	T3-iii	GTCTAGCACCTTGGACCTTGTTGACACTTCTCTCTAGCTT
	L12T	AGGTCCAAGGTGCTAGAC
	T3-iv	TGGTGTCGAACGTTGAAAGGGAGAACTGACTAATACCGATAATTGCGACGAATCACGATTACATGCAGCCTA
	L12F	TTTCAACGTTCGACACCA
	T4-i	GATTCGTCGCAATTATCGCTAGTCTTGGAGTCACACATTG
	T4-ii	CCAAGTAGTCTCAGGTAAAAGTAAGCCGATAATTGCGACGAATC
	T4-iii	GTAATCGGCATCTGTAACAATATTACCTGAGACTACTTGG
	L13T	GTTACAGATGCCGATTAC
	T4-iv	CACAAGGCTTAGAAACAGAGGGAGAACTGACTAATACAGAGGCCCATAGAGTAGACTTCATACCCGTGCTGGC
	L13F	CTGTTTCTAAGCCTTGTG
	O2-i	CGTAAGTTACTCCAGGATACCACTGCATGCCCGTTAGAAA
	O2-ii	CTCAGATAGAAGTCCCTGTGTGATCCTGGAGTAACTTACG
	L1F	CAGGGACTTCTATCTGAG
	O2-iii	GCGTATATGGATAGAGTCGATTTGCCGCTTAGTCTGAGGTGTAGGGTCTCGTAT
	L1T	GACTCTATCCATATACGC
	O3-i	CTGTTTCTAAGCCTTGTGTGATTGGTGTCGAACGTTGAAA
	O3-ii	GTGGTATCCGAGTTCATTTGTTCACAAGGCTTAGAAACAG
	L2F	AATGAACTCGGATACCAC
	O3-iii	TCAACCTGATCATCTGACTAGTCTAGCACCTTGGACCTGTAATCGGCATCTGTAAC
	L2T	GTCAGATGATCAGGTTGA
	O1-i	AATGAACTCGGATACCACCATGCTCAGATAGAAGTCCCTG
	O1-ii	AGTTGTAGCGATTGGTCATTAAGTGGTATCCGAGTTCATT
	ZF	TGACCAATCGCTACAACT
	O1-iii	AGGATACAACACATGGTCGCGTATATGGATAGAGTCTCAACCTGATCATCTGAC
	ZT	GACCATGTGTTGTATCCT
Inputs for ALU	AT	AGGAACCGGCACAGAGAT
	BT	CTTATATCCGCACTCTGG
	AF	CAGTCAGTGGTATTGTCG
	BF	AGGTCCTAGGCTAGCTGT
	CT	ACCAACCGAATATAACGC
	CF	AATTCTAAGCAGCCGTCA
Opcodes for ALU	$S0_F$	CAATGTGTGACTCCAAGA
	$S0_T$	GTATTAGTCAGTTCTCCC
	$S1_F$	GATTCGTCGCAATTATCG
	$S1_T$	TCTACTCTATGGGCCTCT
ALU	N2-i	CAGTCAGTGGTATTGTCGTCGAATCTCTGTGCCGGTTCCT
	N2-ii	ACCCATTAAGCCTAACTGATATCGACAATACCACTGACTG
	L8F	CAGTTAGGCTTAATGGGT

	N2-iii	TATGTGTTCGTGAGTACCCCAGAGTGCGGATATAAGACAGCTAGCCTAGGACCT
	L8T	GGTACTCACGAACACATA
	O6-i	AGGTCCTAGGCTAGCTGTAGTCCCAGAGTGCGGATATAAG
	O6-ii	TCCACTGAAGAACGACCTTGTGACAGCTAGCCTAGGACCT
	L9F	AGGTCGTTCTTCAGTGGA
	O6-iii	GATGGTCTTAGCTTGGTTATCTCTGTGCCGGTTCCTCGACAATACCACTGACTG
	L9T	AACCAAGCTAAGACCATC
	A8-i	AACCAAGCTAAGACCATCGTTGTATGTGTTCGTGAGTACC
	A8-ii	GCATACTTGTGGGTCTTTGTTGGATGGTCTTAGCTTGGTT
	L7T	AAAGACCCACAAGTATGC
	A8-iii	TACTCTCACTCGATCACCACCCATTAAGCCTAACTGCCTCCACTGAAGAACGACCT
	L7F	GGTGATCGAGTGAGAGTA
	N1-i	AAAGACCCACAAGTATGCTCTCGCGTTATATTCGGTTGGT
	N1-ii	CTTCATACCCGTGCTGGCTTTAGCATACTTGTGGGTCTTT
	OPF	GCCAGCACGGGTATGAAG
	O5-i	GGTGATCGAGTGAGAGTATGGTTGACGGCTGCTTAGAATT
	O5-ii	CTTCATACCCGTGCTGGCGTCCTACTCTCACTCGATCACC
	OPF	GCCAGCACGGGTATGAAG
	A5-i	AAAGACCCACAAGTATGCGACCAACCGAATATAACGCAAGATGACGGCTGCTTAGAATTGTACTCTCACTCGATCACC
	A5-ii	TTACCTGAGACTACTTGGACGCGCGTTATATTCGGTTGGTTGCATACTTGTGGGTCTTT
	OPT	CCAAGTAGTCTCAGGTAA
	A6-i	AAAGACCCACAAGTATGCTCTCGCGTTATATTCGGTTGGT
	A6-ii	CTCATCACTCTGGGTCTGCTTCGCATACTTGTGGGTCTTT
	L5T	CAGACCCAGAGTGATGAG
	A7-i	CAGTCAGTGGTATTGTCGTCCTATCTCTGTGCCGGTTCCT
	A7-ii	GACACTTCTCTCTAGCTTTCCTCGACAATACCACTGACTG
	L6T	AAGCTAGAGAGAAGTGTC
	A7-iii	ACGATTACATGCAGCCTACCAGAGTGCGGATATAAGACAGCTAGCCTAGGACCT
	L6F	TAGGCTGCATGTAATCGT
	O4-i	TAGGCTGCATGTAATCGTTGTGTGACGGCTGCTTAGAATTGTACTCTCACTCGATCACC
	O4-ii	ACTCACATTCCTGGAATCATTCACGATTACATGCAGCCTA
	COF	GATTCCAGGAATGTGAGT
	O4-iii	CGGTCCAATTAGCTCAAACCTCATCACTCTGGGTCTGGACACTTCTCTCTAGCTT
	COT	TTTGAGCTAATTGGACCG
	T1-i	TCTACTCTATGGGCCTCTTCTCGGGAGAACTGACTAATAC
	T1-ii	GGTACTCACGAACACATATTATTCTTAGAGGCCCATAGAGTAGA
	T1-iii	GATTTGCCGCTTAGTCTGCCTTTATGTGTTCGTGAGTACC
	L10T	CAGACTAAGCGGCAAATC

	T1-iv	CTGCATGCCCGTTAGAAATCTTGGAGTCACACATTGCGATAATTGCGACGAATCACCCATTAAGCCTAACTG
	L10F	TTTCTAACGGGCATGCAG
	T2-i	GATTCGTCGCAATTATCGGTGTGGGAGAACTGACTAATAC
	T2-ii	AACCAAGCTAAGACCATCTGTGTGTGCGATAATTGCGACGAATC
	T2-iii	AGGTGTAGGGTCTCGTATAGTCGATGGTCTTAGCTTGGTT
	L11T	ATACGAGACCCTACACCT
	T2-iv	ATCCTGGAGTAACTTACGTCTTGGAGTCACACATTGAGAGGCCCATAGAGTAGAAGTCCACTGAAGAACGACCT
	L11F	CGTAAGTTACTCCAGGAT
	T3-i	TCTACTCTATGGGCCTCTTTAATCTTGGAGTCACACATTG
	T3-ii	AAGCTAGAGAGAAGTGTCGGTTGGTTAGAGGCCCATAGAGTAGA
	T3-iii	GTCTAGCACCTTGGACCTTGTTGACACTTCTCTCTAGCTT
	L12T	AGGTCCAAGGTGCTAGAC
	T3-iv	TGGTGTCGAACGTTGAAAGGGAGAACTGACTAATACCGATAATTGCGACGAATCACGATTACATGCAGCCTA
	L12F	TTTCAACGTTCGACACCA
	T4-i	GATTCGTCGCAATTATCGCTAGTCTTGGAGTCACACATTG
	T4-ii	CCAAGTAGTCTCAGGTAAAAGTAAGCCGATAATTGCGACGAATC
	T4-iii	GTAATCGGCATCTGTAACAATATTACCTGAGACTACTTGG
	L13T	GTTACAGATGCCGATTAC
	T4-iv	CACAAGGCTTAGAAACAGAGGGAGAACTGACTAATACAGAGGCCCATAGAGTAGACTTCATACCCGTGCTGGCT
	L13F	CTGTTTCTAAGCCTTGTG
	O2-iii	AGGATACAACACATGGTCGATTTGCCGCTTAGTCTGAGGTGTAGGGTCTCGTAT
	YT	GACCATGTGTTGTATCCT
	O3-iii	AGGATACAACACATGGTCGTCTAGCACCTTGGACCTGTAATCGGCATCTGTAAC
	YT	GACCATGTGTTGTATCCT
	O1-i	CGTAAGTTACTCCAGGATACCACTGCATGCCCGTTAGAAA
	O1-ii	TTTCAACGTTCGACACCATAGCTGAGATCCTGGAGTAACTTACG
	O1-iii	CTGTTTCTAAGCCTTGTGTGATTGATTGGTGTCGAACGTTGAAA
	O1-iv	AGTTGTAGCGATTGGTCATGTTCACAAGGCTTAGAAACAG
	YF	TGACCAATCGCTACAACT
TRI	TR-i	(FAM)-CCGCCTTTCTACAGATCCGATTACCTGAGACTACTTGG
	TR-ii	GGATCTGTAGAAAGGCGG-(BHQ-1)
FRI	FR-i	(ROX)-CGTTTCAGGCTAGAGAACTCCTTCATACCCGTGCTGGC
	FR-ii	GTTCTCTAGCCTGAAACG-(BHQ-2)

TRII	TR-iii	(HEX)-ACACCCTCAGCGCTAGTAGTCGGTCCAATTAGCTCAAA
	TR-iV	TACTAGCGCTGAGGGTGT-(BHQ-1)
FRII	FR-iii	(Cy5)-CCAGTACATCAGATATAGTCACTCACATTCCTGGAATC
	FR-iV	CTATATCTGATGTACTGG-(BHQ-2)
TRIII	TR-V	(FAM)-TGGCATAACATCGCGCTCTTAGGATACAACACATGGTC
	TR-Vi	GAGCGCGATGTTATGCCA-(BHQ-1)
FRIII	FR-V	(ROX)-GAGGAGGTCGTAATTGGCTTAGTTGTAGCGATTGGTCA
	FR-Vi	GCCAATTACGACCTCCTC-(BHQ-2)

References

1 Qian, L. & Winfree, E. Scaling up digital circuit computation with DNA strand displacement cascades. *Science* **332**, 1196-1201, (2011).

2 Li, W., Zhang, F., Yan, H. & Liu, Y. DNA based arithmetic function: a half adder based on DNA strand displacement. *Nanoscale* **8**, 3775-3784, (2016).

3 Song, T., Garg, S., Mokhtar, R., Bui, H. & Reif, J. Design and Analysis of Compact DNA Strand Displacement Circuits for Analog Computation Using Autocatalytic Amplifiers. *ACS Synth. Biol.* **7**, 46-53, (2018).
